# Reactivity and Magnetic Coupling of Triangulene Dimers Linked via *para*‐Biphenyl Units

**DOI:** 10.1002/anie.202501874

**Published:** 2025-02-28

**Authors:** Elena Pérez‐Elvira, Marco Lozano, Qiang Huang, Ji Ma, Aurelio Gallardo, Ana Barragán, Koen Lauwaet, José M. Gallego, Rodolfo Miranda, Pavel Jelínek, David Écija, Diego Soler‐Polo, Xinliang Feng, José I. Urgel

**Affiliations:** ^1^ IMDEA Nanoscience C/ Faraday 9, Campus de Cantoblanco 28049 Madrid Spain; ^2^ Institute of Physics of the Czech Academy of Science CZ-16253 Praha Czech Republic; ^3^ Center for Advancing Electronics Dresden (cfaed) & Faculty of Chemistry and Food Chemistry Technische Universität Dresden 01069 Dresden Germany; ^4^ College of Materials Science and Optoelectronic Technology & Center of Materials Science and Optoelectronics Engineering University of Chinese Academy of Science 100049 Beijing P. R. China; ^5^ Max Planck Institute of Microstructure Physics Weinberg 2 06120 Halle Germany; ^6^ Instituto de Ciencia de Materiales de Madrid (ICMM), CSIC Cantoblanco 28049 Madrid Spain; ^7^ Unidad de Nanomateriales avanzados Imdea Nanoscience, Unidad asociada al CSIC por el ICMM 28049 Madrid Spain; ^8^ Regional Centre of Advanced Technologies and Materials Palacký University Olomouc 771 46 Olomouc Czech Republic

**Keywords:** π-electron magnetism, surface chemistry, triangulenes, scanning tunneling microscopy, open-shell character

## Abstract

Triangulene and its homologues are promising building blocks for high‐spin low‐dimensional networks with long‐range magnetic order. Despite the recent progress in the synthesis and characterization of coupled triangulenes, key parameters such as the number of organic linking units or their dihedral angles remain scarce, making further studies crucial for an essential understanding of their implications. Here, we investigate the synthesis and reactivity of two triangulene dimers linked by two (**Dimer 1**) or one (**Dimer 2**) *para*‐biphenyl units, respectively, on a metal surface in an ultra‐high vacuum environment. First‐principles calculations and model Hamiltonians reveal how spin excitation and radical character depend on the rotation of the *para*‐biphenyl units. Comprehensive scanning tunneling microscopy (STM) in combination with density functional theory (DFT) calculations confirm the successful formation of **Dimer 1** on Au(111). Non‐contact atomic force microscopy (nc‐AFM) measurements resolve the twisted conformation of the linking *para*‐biphenyl units for **Dimer 1**. On the contrary, the inherent flexibility of **Dimer 2** induces the planarization of the *para*‐biphenyl, resulting in the spontaneous formation of two additional five‐membered rings per dimer connected by a single C−C bond (**Dimers 2′**). Furthermore, scanning tunneling spectroscopy (STS) measurements confirm the antiferromagnetic (S=0) coupling of the observed dimers, underscoring the critical influence of dihedral angles and structural flexibility of the linking units in π‐electron magnetic nanostructures.

## Graphical abstract

Triangulene dimers linked by one or two *para*‐biphenyl units were synthesized on Au(111). STM, non‐contact AFM, and scanning tunneling spectroscopy measurements reveal an open‐shell singlet magnetic exchange coupling for Dimer 1 (*E*_S‐T_=15 mV), influenced by the dihedral angles of the two *para*‐biphenyl units. Dimers 2′ with an *E*_S‐T_=6.6 mV were obtained from a more flexible precursor equipped with one *para*‐biphenyl linker.

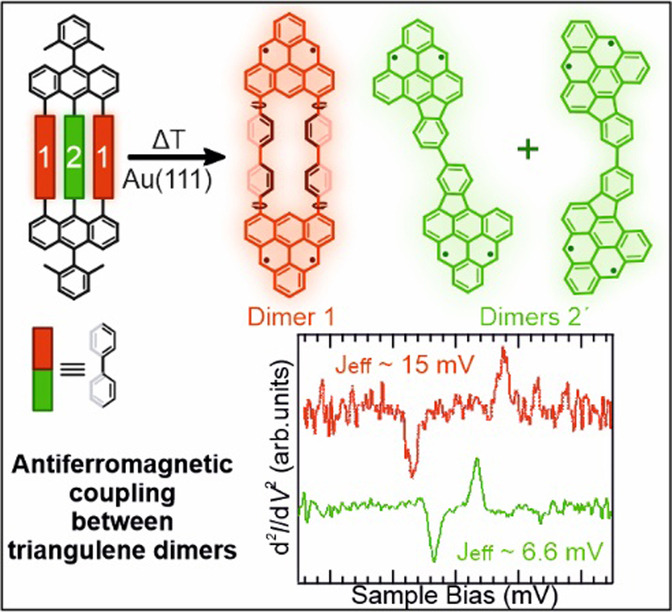

[3]Triangulene, one of the smallest non‐Kekulé graphene fragments, consists of six fused benzenoid rings with three zigzag edges. It is one of the most studied fundamental open‐shell (magnetic) π‐conjugated fragments with potential for modern carbon‐based spintronic devices.[^1^, ^2^, ^3^] Magnetism in triangulene arises from an intrinsic sublattice asymmetry in its bipartite honeycomb lattice. This results in a high spin ground state (S=1) with two unpaired π‐electrons localized at two non‐bonding molecular orbitals.[^4^, ^5^] Its inherent reactivity that arises from their unpaired electrons has hampered the solution synthesis of unsubstituted triangulene fragments.^[6]^ Only recently, their synthesis confined on surfaces following a bottom‐up approach under an ultrahigh vacuum (UHV) environment has appeared as a novel method for the synthesis and characterization of such highly reactive graphene fragments.

The pioneering synthesis of [3]triangulene in 2017 by Pavliček et al. on insulating and metallic surfaces^[7]^ marked the starting box for the fabrication of triangulene derivatives. Since then, a plethora of studies related to different members of this family have been reported,[^8^, ^9^, ^10^, ^11^] including the effects of atomic doping[^10^, ^12^, ^13^, ^14^, ^15^, ^16^] which also enables the precise tuning of their magnetic properties. Contemporarily, carbon‐carbon coupling reactions, together with the incorporation of organic linkers between triangulene fragments, not only adjusts the magnetic exchange coupling (MEC) between units but also modulates magnetic correlations, allowing for the control of high‐ or low‐spin ground states.[^17^, ^18^, ^19^, ^20^, ^21^, ^22^, ^23^] Despite all the progress made in recent years, the crucial role of the number of organic linkers and their dihedral angles might directly affect orbital overlap. Thus, they have an influence on the MEC between coupled triangulenes, which remains clearly underexplored.[^24^, ^25^, ^26^] In fact, these aspects could also be relevant for the study of triangulene‐based organic quantum spin chains,[^27^, ^28^] open‐shell nanographenes,[^29^, ^30^, ^31^, ^32^, ^33^, ^34^] and not yet realized two‐dimensional lattices and networks.^[35]^

In this communication, we introduce the exemplary investigation of triangulene dimers interconnected by two *para*‐biphenyl units through unconventional linking sites and one *para*‐biphenyl unit linked through the α‐position.^[6]^

**Dimer 1** (triangulene dimer interconnected by two *para*‐biphenyl units) was experimentally achieved via surface‐assisted oxidative ring‐closure of predesigned precursor **1** on a coinage metal surface under ultra‐high vacuum (UHV) conditions. Its chemical structure was evidenced by scanning tunneling microscopy (STM), and non‐contact atomic force microscopy (nc‐AFM). Additionally, scanning tunneling spectroscopy (STS) measurements, complemented by density‐functional theory (DFT) and many‐body complete active space configuration interaction (CAS‐CI) calculations, provide detailed insights into the electronic structure. However, the expected formation of **Dimer 2** from precursor **2** featuring one *para*‐biphenyl linker was hampered due to an increased flexibility with respect to **1**, which induces planarization through the unforeseen formation of two five‐membered rings between triangulenes and the *para*‐biphenyl unit (**Dimer 2′A** and **B**), as revealed by STM images (see Scheme 1 for the solution synthesis of precursors **1** and **2**, and the on‐surface generation of **Dimer 1**, **2′A** and **2′B**).

**Scheme 1:**
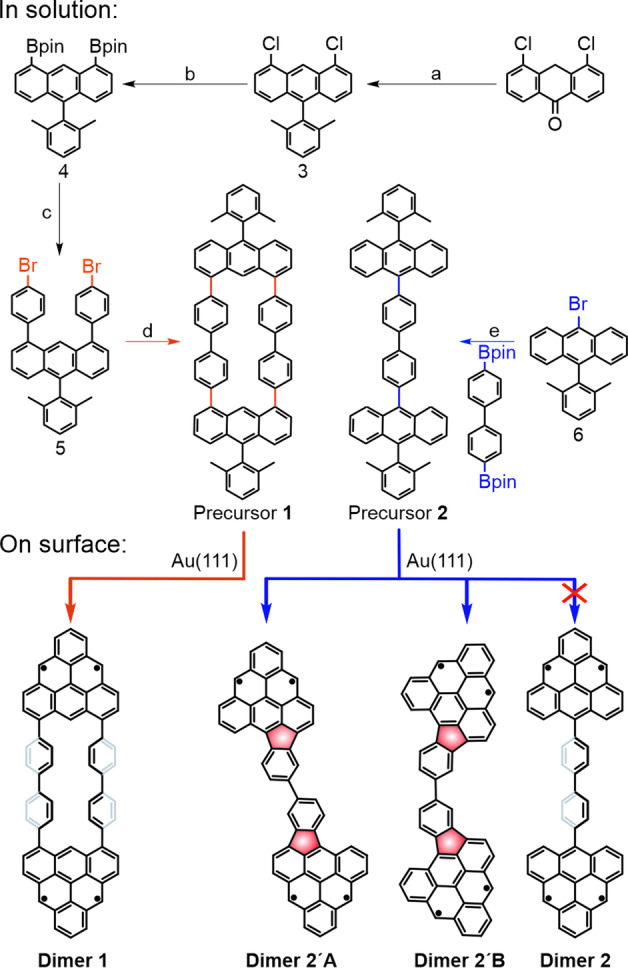
Synthesis of triangulene dimers reported in this work. Reagents and conditions for the solution synthesis of precursors **1** and **2**: a. (2,6‐dimethylphenyl)magnesium bromide, Et_2_O, rt, 12 h, 46 %; b. Bis(pinacolato)diboron (B_2_pin_2_), SPhos Pd G3, KOAc, DMF, 130 °C, 48 h, 35 %; c. Pd(PPh_3_)_4_, 1,4‐dibromobenzene, K_2_CO_3_, toluene/H_2_O, 80 °C, 24 h, 59 %; d. Ni(cod)_2_, 2,2′‐bipyridyl,1,5‐cyclooctadien (cod), toluene, 80 °C, 48 h, 12 %; e. Pd(PPh_3_)_4_, K_2_CO_3_, toluene/H_2_O, 80 °C, 24 h, 60 %. **Dimers 1**, **2′A** and **2′B** are obtained after sublimation of precursors **1** and **2** on an Au(111) surface kept at 200 °C.

We first theoretically studied the spin excitation and radical character of **Dimers 1** and **2** as a function of the *para*‐biphenyl angles by First‐principles calculations and model Hamiltonian studies. To afford a complete exploration of the parameter space, we employ the Hubbard model with parameters t=‐2.8 eV and U=3.8.^[36]^ This performs excellently for hydrocarbons, as seen in many reported studies.[^29^, ^30^, ^37^] To account for the benzene rotation, we modify the hopping (t) between the carbon atoms according to the overlap of p_z_ orbitals, t(α)=‐2.8 cos(α) as a function of dihedral angle α, see Schemes in Figure 1a,b. At the many‐body level (CAS‐CI), this yields the spin‐excitation maps shown in Figure 1a,b. Each triangulene possesses two ferromagnetically coupled radicals, but the connection results in a partial quenching of the radical character and an open‐shell singlet ground state for all the configurations. When the *para*‐biphenyl units of a branch are at 90° with respect to the triangulene units, the π‐systems are disconnected and the singlet, triplet and quintet states are degenerated. As shown in Figure 1a, for **Dimer 1** there are two independent channels through which the triangulenes connect. We rationalize these trends with a four‐site spin model (see Figure 1c). The sites of the radicals inside each triangulene have a fixed large ferromagnetic coupling (J=400 meV), corresponding to the large spin excitation of the S=1 triangulene. The inter‐triangulene couplings are all antiferromagnetic, depend on the angles through the overlaps (see Supporting Information for details), and reach their maximum values for the planar structures (35 and 18 meV, respectively for **Dimer 1** and **Dimer 2**). Interestingly, this simple spin model captures the full behavior of these systems (see Figure S1, which shows the spin excitation maps calculated from the spin model).

**Figure 1:**
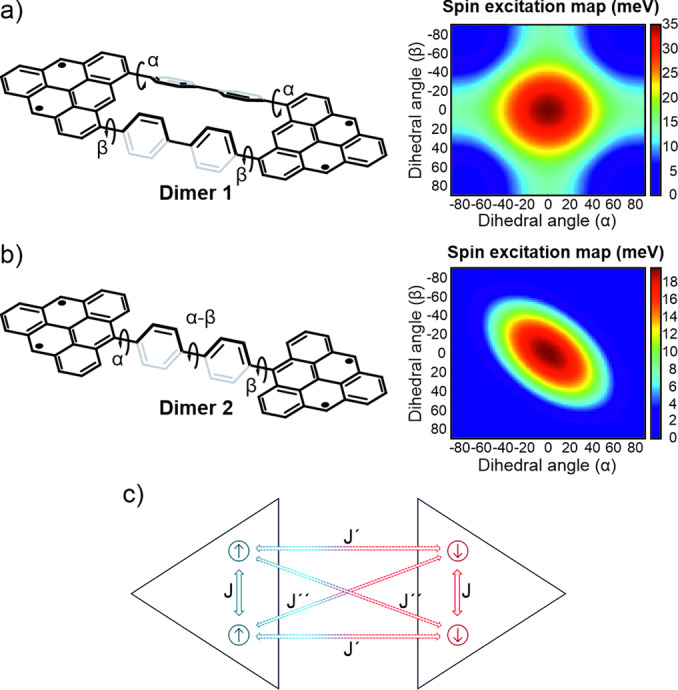
Influence of the *para*‐biphenyl dihedral angles in the π‐electron magnetism of triangulene dimers. a,b) Geometries and maps of the singlet‐triplet excitation energy for the triangulenes coupled through two (a) and one (b) *para*‐biphenyl channels, calculated from CAS‐CI(4,4) for the Hubbard model. c) Effective spin model that reproduces the spectra of the systems. J is ferromagnetic and is fixed by the spin‐excitation of the triangulenes, whereas J’ and J’’ are antiferromagnetic and parametrized by the overlap of p_z_ orbitals. The two triangles represent both [3]triangulene units.

Motivated by our theoretical predictions, we next focused on the syntheses of both triangulene dimers and their comprehensive structural and electronic characterization. To this end, molecular precursors **1** and **2** were synthesized in solution (Scheme 1). First, commercially available 4,5‐dichloroanthracen‐9(10*H*)‐one was reacted with an excess of (2,6‐dimethylphenyl)magnesium bromide to afford compound **3**. Next, a Miyaura borylation reaction between **3** and bis(pinacolato)diboron was carried out in DMF at 130 °C, yielding compound **4**. Subsequently, a Suzuki coupling between **4** and 1,4‐dibromobenzene produced 1,8‐bis(4‐bromophenyl)‐10‐(2,6‐dimethylphenyl)anthracene (**5**) in 59 % yield. In the last step, a nickel‐catalyzed Yamamoto coupling of **5** resulted in the formation of precursor **1** in 12 % yield. Additionally, a Pd‐catalyzed Suzuki coupling between 9‐bromo‐10‐(2,6‐dimethylphenyl)anthracene (**6**) and commercially available 4,4′‐bis(4,4,5,5‐tetramethyl‐1,3,2‐dioxaborolan‐2‐yl)‐1,1′‐biphenyl afforded precursor **2** with a yield of 60 % (see Supporting Information for synthetic and characterization details).

Sublimation of **1** onto a clean Au(111) surface held at 200 °C affords the oxidative ring‐closure reaction, i.e. formation of **Dimer 1** (see Figure 2a), coexisting with dimers with unreacted methyl groups and ill‐defined structures (Figure S2). Constant‐current and constant‐height STM images (Figure 2b,c, respectively) reveal the presence of two connected triangular‐shaped molecular species on the surface. Herein, two distinguishable apparent heights of 1.4 Å at the triangular edges and 2.0 Å at the central connection (measured at a sample bias of −1.3 V, Figure 2b) are discerned. To confirm the chemical structure of **Dimer 1**, we performed nc‐AFM measurements using a carbon monoxide (CO)‐functionalized tip.^[38]^ Figure 2d depicts the resulting constant‐height frequency‐shift image. The non‐planarity of the central segment of the molecule, which arises due to the intramolecular H−H steric hindrance,^[39]^ is clearly manifested. Such non‐planarity is reproduced in the simulated nc‐AFM image using the PP‐SPM code^[40]^ depicted in Figure 2e (see Figure S3 for a constant‐height frequency shift nc‐AFM image of **Dimer 1** acquired at a closer distance). We relax the structure of the molecule on Au(111), as shown in Figure 2f, at the DFT‐PBE level of theory with the FH‐AIMS code.^[41]^ A tilt of the *para*‐biphenyl units that link both triangulenes is distinguished, with a dihedral angle (Θ) of ~34° for the four benzene rings with respect to the underlying surface (notice that the experimental value cannot be determined by nc‐AFM). Additionally, we calculated various ring alternation patterns for the inner benzene rings (Figure S4), but none of these geometries matched the experimentally observed adsorption geometry of **Dimer 1**.

**Figure 2:**
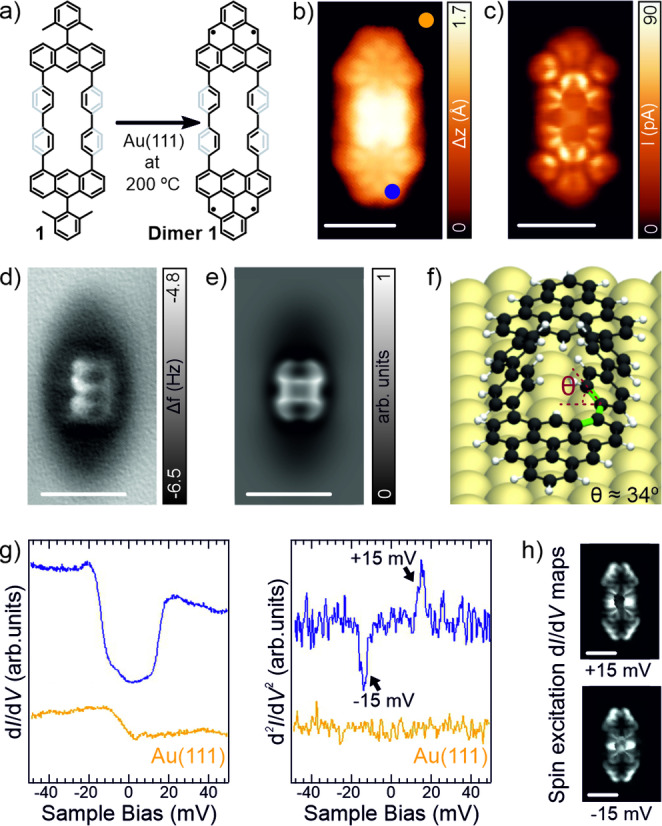
Structural and electronic characterization of **Dimer 1** synthesized on Au(111). a) On‐surface synthesis of **Dimer 1** reported in this work. b,c) Constant‐current (b) and constant‐height (c) high‐resolution STM images of a two connected triangular‐shape molecular species assigned to **Dimer 1**. Scanning parameters: (b) *V*_b_=‐1.3 V, *I*_t_=500 pA, (c) *V*_b_=5 mV, *I*_t_=50 pA. d) Constant‐height frequency‐shift nc‐AFM image of panel b acquired with a CO‐functionalized tip (z offset of +250 pm below STM set point: 5 mV, 50 pA). e) Simulated nc‐AFM image of panel d. f) DFT equilibrium geometry of the molecule on Au(111). Bonds highlighted in green define the dihedral angle Θ, which corresponds to the angle between the plane containing the carbon atoms of the triangulene units adsorbed parallel to the gold surface and the other plane containing the tilted carbon atoms of the four *para*‐biphenyl units. g) Differential conductance spectra and the corresponding inelastic electron tunnelling spectroscopy (IETS) spectra (open feedback parameters: *V*_b_=50 mV, *I*_t_=500 pA and V_rms_=1.6 mV) from where the value of E_S‐T_ is deduced. Acquisition positions for the spectra are indicated by color rounded marks in panel b. h) Constant‐current d*I*/d*V* maps of the molecule at its spin excitation energies (*I*_t_=150 pA and V_rms_=1.6 mV). All scale bars=1 nm.

Next, we investigate the electronic structure of **Dimer 1** by STS measurements. Figure 2g displays the high‐resolution differential conductance d*I*/d*V* vs. *V* spectra, where *I* and *V* denote the tunneling current and bias voltage. We observe two steps in conductance symmetric around the Fermi energy, often indicative of inelastic excitations, with energetic positions at roughly ±15 meV as observed in the d^2^ *I*/d*V* ^2^ spectroscopy (see Figure S5, where the fitting of the spectrum from Figure 2g is shown). We ascribe the d*I*/d*V* steps to singlet‐triplet spin excitations that define the MEC between triangulene units with a E_S‐T_~15 mV, in good agreement with the map of the singlet‐triplet excitation energy for **Dimer 1** with Θ=α=β=34°, calculated from CAS‐CI(4,4) for the Hubbard model (theoretical E_S‐T_=20 meV). Figure 2h also shows the spatially‐resolved d*I*/d*V* maps at the spin excitation thresholds, which are in line with the simulated spin excitation signal of **Dimer 1** (see Figure S6). We have also performed long‐range d*I*/d*V* spectra featuring a series of prominent peaks in the local density of states (LDOS, Figure S7). We compared the experimental d*I*/d*V* maps acquired at these energetic positions with the calculated d*I*/d*V* maps^[42]^ of the molecule obtained from Dyson orbitals^[43]^ for the adding and removal of charge processes. Based on this comparison, we assign the observed features to positive and negative ion resonances (PIR and NIR), extracting the experimental electronic gaps (Figure S7). We chose this notation due to the almost pure tetra radical character of **Dimer 1**, which highlights the multi‐reference nature of the system, which cannot be described correctly by single‐determinant canonical DFT orbitals.

Surprisingly, sublimation of **2** onto the same surface and preparation conditions as the ones employed to obtain **Dimer 1** did not give rise to the formation of the predicted **Dimer 2**. Instead, we observed the systematic formation of two different triangular‐shape nanostructures (see chemical sketch in Figure 3a), coexisting with some fused species (Figure S8a). Figure 3b,c show the constant‐current STM images corresponding to both type of molecules (**Dimer 2′**). More interestingly, the chemical structure of both **Dimers 2′** is clearly unveiled in the constant‐height acquired with a CO‐functionalized tip. Herein, we observe the formation of two five‐membered rings per dimer, together with the expected oxidative ring closure reaction that promotes the formation of the triangulene units (Figure 3d,e). Additionally, **Dimers 2′** present chiral (**Dimer 2′A**) and achiral (**Dimer 2′B**) adsorption configurations on the surface, without finding a preference for any of them (see Figure S8).^[44]^ Following the structural characterization of **Dimers 2′**, we have focused our attention on their electronic structure. Similar to the electronic structure of **Dimer 1**, the d*I*/d*V* vs. *V* spectra performed on **Dimers 2′** show steps in conductance symmetric around the Fermi energy, this time with energetic positions at roughly ±6.6 meV as observed in the d^2^ *I*/d*V* ^2^ spectroscopy of both dimers (Figure 3f). These singlet‐triplet spin excitations imply a significant decrease of the MEC between triangulene units (E_S‐T_ ~6.6 mV, see fittings of the spectra from Figure 3f in Figure S5) with respect to **Dimer 1**, being in very good agreement with calculations (theoretical E_S‐T_=6.0 mV from CAS‐CI(4,4) for the Hubbard model). Notably, these nanostructures are planar, reducing the dihedral angles of the central benzene rings to 0°, which, in principle, should maximize the MEC of the molecules (see Figure 1b). However, we attribute the observed reduction of the MEC to the presence of the five‐ membered rings in both dimers. These rings preserve the radical character of the molecule while creating an area of increased charge density that leads to a weaker antiferromagnetic coupling (see Figure S9 for the analysis of the Natural Orbitals extracted from the CAS‐CI calculation of all the discussed dimers). Finally, long‐range d*I*/d*V* spectra and the comparison with the DFT‐calculated d*I*/d*V* maps are shown in Figure S10.

**Figure 3:**
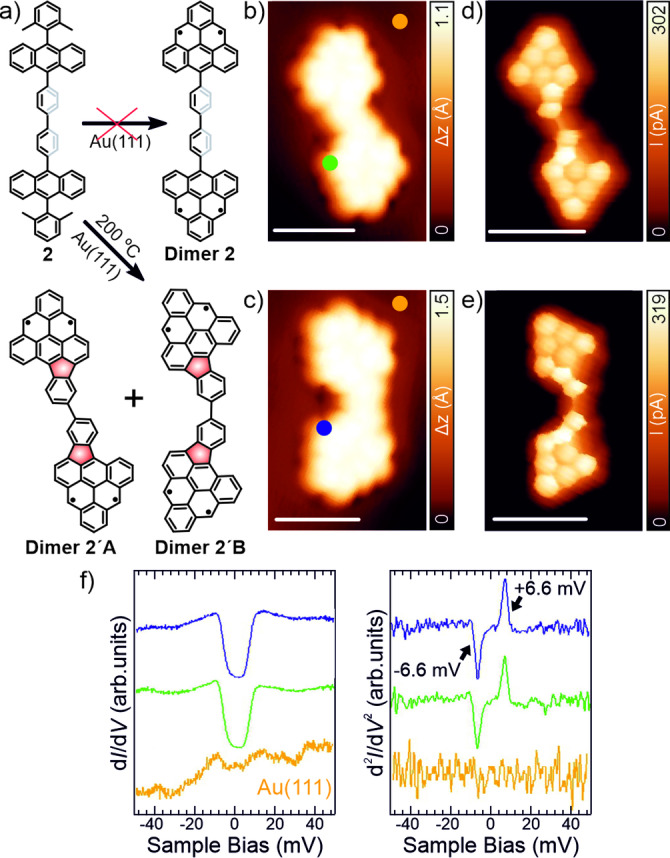
Structural and electronic characterization of **Dimers 2′** synthesized on Au(111). a) Chemical sketch of the on‐surface synthesis of both **Dimers 2′**. b,c) Constant‐current high‐resolution STM images of both dimers. Scanning parameters: *V*_b_=‐0.05 V, *I*_t_=500 pA. d,e) Constant‐height STM images of panel b and c, respectively, acquired with a CO‐functionalized tip. Scanning parameters: *V*_b_=5 mV, *I*_t_=50 pA. f) Short‐range differential conductance spectra and the corresponding inelastic electron tunnelling spectroscopy (IETS) spectra acquired with a CO‐functionalized tip (open feedback parameters: *V*_b_=‐50 mV, *I*_t_=1 nA and V_rms_=0.8 mV) from where the value of E_S‐T_ is deduced. Acquisition positions for the spectra are indicated by color rounded marks in panels b and c. All scale bars=1 nm.

In summary, our study shows the detailed synthesis and characterization of triangulene dimers linked by *para*‐biphenyl units on Au(111). Advanced computational methods demonstrate that variations in the dihedral angle of *para*‐biphenyl linkers can substantially modulate the MEC, revealing a critical parameter for controlling magnetic interactions in carbon‐based systems. **Dimer 1** has been achieved via surface‐assisted oxidative ring closure after thermal annealing of precursor **1** and its structure has been clearly elucidated by STM and nc‐AFM. STS measurements, complemented with theoretical calculations, demonstrate that **Dimer 1** presents an antiferromagnetic coupling between triangulene units (E_S‐T_ ~15 meV), exceeding that of previously studied triangulene dimers. **Dimer 2** could not be achieved, as a result of an increased flexibility and reactivity of precursor **2**, which led to the unexpected formation of two five‐membered rings per molecule through cyclodehydrogenation of the benzene rings of the *para*‐biphenyl unit. We observe two chiral and achiral planar dimers (**Dimers 2′**), with a very similar reduction of the antiferromagnetic coupling (from E_S‐T_~15 meV to E_S‐T_~6.6 meV) with respect to the one expected for the planar **Dimer 2**, which is attributed to the effect of the five‐member rings on the π‐conjugation. These insights emphasize the importance of the interplay between molecular reactivity, flexibility, and magnetic properties of molecular systems, contributing to the design of polyradical systems with potential for spintronic applications.

## Conflict of Interests

The authors declare no conflict of interest.

## Supporting information

As a service to our authors and readers, this journal provides supporting information supplied by the authors. Such materials are peer reviewed and may be re‐organized for online delivery, but are not copy‐edited or typeset. Technical support issues arising from supporting information (other than missing files) should be addressed to the authors.

Supporting Information

## Data Availability

The data that support the findings of this study are available from the corresponding author upon reasonable request.
