# Supplementary material for: Reactivity and Magnetic Coupling of Triangulene Dimers Linked via para‐Biphenyl Units
Source: Angew Chem Int Ed Engl. 2025 Feb 28;64(17):e202501874. doi: 10.1002/anie.202501874 (PMC12015394; doi:10.1002/anie.202501874)
Supplement: Supplementary file 1 — Supporting Information [file ANIE-64-e202501874-s001.pdf]

Angewandte Chemie
Eine Zeitschrift der Gesellschaft Deutscher Chemiker

Supporting Information

Reactivity and Magnetic Coupling of Triangulene Dimers Linked via *para*-Biphenyl Units

E. Pérez-Elvira, M. Lozano, Q. Huang, J. Ma, A. Gallardo, A. Barragán, K. Lauwaet, J. M. Gallego, R. Miranda, P. Jelínek, D. Écija*, D. Soler-Polo*, X. Feng, J. I. Urgel**

Supporting Information
©Wiley-VCH 2021
69451 Weinheim, Germany

Reactivity and Magnetic coupling of Triangulene Dimers linked via *para*-Biphenyl Units

Elena Pérez-Elvira,†,[a] Marco Lozano,†,[b] Qiang Huang, †,[c] Ji Ma,*[d,e] Aurelio Gallardo,[a] Ana Barragán,[a] Koen Lauwaet,[a] José M. Gallego,[f] Rodolfo Miranda,[a] Pavel Jelínek,[b,h] David Écija,*[a,g] Diego Soler-Polo,*[b] Xinliang Feng,[c,e] and José I. Urgel*[a,g]

[a] Elena Pérez-Elvira, Dr. Ana Barragán, Dr. Koen Lauwaet, Prof. Rodolfo Miranda, Prof. David Écija and Dr. José I. Urgel
IMDEA Nanoscience, C/ Faraday 9, Campus de Cantoblanco, 28049 Madrid, Spain
E-mail: david.ecija@imdea.org, jose-ignacio.urgel@imdea.org
[b] Marco Lozano, Dr. Diego Soler-Polo and Prof. Pavel Jelínek
Institute of Physics of the Czech Academy of Science, CZ-16253 Praha, Czech Republic
E-mail: jelinekp@fzu.cz
[c] Dr. Qiang Huang, Prof. Xinliang Feng
Center for Advancing Electronics Dresden (cfaed) & Faculty of Chemistry and Food Chemistry, Technische Universität Dresden, 01069 Dresden, Germany
[d] Dr. Ji Ma
College of Materials Science and Optoelectronic Technology & Center of Materials Science and Optoelectronics Engineering, University of Chinese Academy of Science, 100049 Beijing, P. R. China
E-mail: maji@ucas.ac.cn
[e] Dr. Ji Ma, Prof. Xinliang Feng
Max Planck Institute of Microstructure Physics, Weinberg 2, 06120 Halle, Germany
[f] Dr. José M. Gallego
Instituto de Ciencia de Materiales de Madrid (ICMM), CSIC, Cantoblanco, 28049 Madrid, Spain
[g] Prof. David Écija and Dr. José I. Urgel
Unidad de Nanomateriales avanzados, Imdea Nanoscience, Unidad asociada al CSIC por el ICMM, 28049 Madrid, Spain
[h] Prof. Pavel Jelínek
Regional Centre of Advanced Technologies and Materials, Palacký University Olomouc, 771 46 Olomouc, Czech Republic

† Equally contributing authors

Supporting information and the ORCID identification number(s) forthe author(s) of this article can be found under:

https://doi.org

2020 The Authors. Published by Wiley-VCH Verlag GmbH&Co. KGaA. This is an open access article under the terms of the Creative Commons Attribution-Non Commercial License, which permits use, distribution and reproduction in any medium, provided the original work is properly cited and is not used for commercial purposes

Table of Contents

1. **Experimental and computational methods**
2. **Additional experimental and computational results**
3. **General methods and materials**
4. **Synthetic procedures and characterization data**
5. **References**

1. Experimental and computational methods

1.1 Experimental methods

The experiments were conducted at IMDEA Nanoscience (Madrid, Spain) in a custom-designed ultra-high vacuum system with a base pressure below 2.0 × 10^{-10} mbar, which hosted a commercial low-temperature microscope with STM/AFM capabilities from Scienta Omicron. The Au(111) substrate was prepared by Ar^+ sputtering (E = 1.5 keV) followed by annealing at 740 K for 10 minutes. Molecular precursor **1** and **2** were sublimed on a clean Au(111) surface by thermal evaporation (Kentax TCE-BSC. $T_{sublimation}$ **1** = 300 ºC and $T_{sublimation}$ **2** = 250 ºC) with a typical deposition rate of 0.3 Å/min at room temperature, controlled by a quartz microbalance (LewVac). The sample temperature during deposition of **1** was 200 °C, which led to the oxidative ring-closure reaction; while for **2** a subsequent annealing at 250 ºC was necessary to induce the surface reactions.

STM images and d*I*/d*V* maps were acquired in constant-current mode with tungsten tips that were electrochemically etched, at a sample temperature of 4.3 K (LakeShore). Both d*I*/d*V* - and spin excitation maps and curves were obtained using the lock-in technique (SR830). High-resolution STM images were acquired by recording the current channel and using CO-functionalized tips[1] to scan **Dimer 1** and **Dimers 2´** in constant-height mode. The figure captions indicate the open feedback settings and subsequent tip approach distance (Z_{offset}) for each image. The scanning parameters used for each figure are described in the figure captions.

Non-contact AFM measurements were performed with a tungsten tip attached to a Qplus tuning fork sensor from Omicron.[2] The tip was then functionalized by a controlled adsorption of a single CO molecule as previously described. The functionalized tip enables the imaging of the intramolecular structure of organic molecules.[3] The sensor was driven at its resonance frequency (26 kHz for Qplus) with a constant amplitude of ~60 pm. The shift in the resonance frequency of the sensor (with the attached CO-functionalized tip) was recorded in a constant-height mode (Omicron Matrix electronics and MFLi PLL by Zurich Instruments for Omicron).

To investigate the spin-excitation properties of the system, we performed fits on second derivative $d^2I/dV^2(V)$ spectra. The system under study is anticipated to exhibit a non-magnetic S=0 ground state, with the potential for excitations to an S = 1 state. Building on the theoretical framework outlined in ref.,[4] which primarily addresses the conductance (first derivative) of such systems, we implemented the analytical derivative to fit our IETS spectra. To account for both intrinsic and instrumental sources of line broadening, the temperature was left as a free parameter in the fit, following the suggestion in ref.[5]. All fits were performed using Python, utilizing the lmfit library to implement non-linear least squares optimization, and the access2theMatrix package (https://pypi.org/project/access2theMatrix/) for loading the spectra. The STM and nc-AFM images, together with the STS measurements were analyzed using WSxM 5.0.[6]

1.2 Computational details

For the optimization of the four considered absorption structures in Figure S2, we used density functional theory simulations using the FHI-AIMS package.[7] We modeled the metallic substrate using a 3-layered Au(111) substrate with a 12x8 supercell, with periodic boundary conditions, a lattice constant of $a = 4.0782$ Å and at least 20 Å of vacuum in the $\hat{z}$ direction. During the optimization, the coordinates of the two topmost layers of gold where in all cases allowed to relax.

The convergence parameters for forces and total energy where set at $10^{-2} eV/Å$ and $10^{-5} eV$ and the reciprocal space was described sampling only the gamma point of the Brillouin zone. All the simulations were performed at the GGA-PBE level of theory[8] and the Tkatchenko-Scheffler treatment of the van der Waals interactions.[9]

The AFM simulations were done using the Probe-Particle Model[10] with a stiffness parameter of the CO probe of $0.24\, N \cdot m^{-1}$ and a charge correction coefficient of $-0.1\, e$. The tip-sample electrostatic interactions were included using the DFT calculated Hartree potential of the sample.

2. Additional experimental and computational results

Theoretical dI/dV maps

Theoretical dI/dV maps were calculated by the Probe Particle Scanning Probe Microscopy (PP-SPM) model for a CO-like tip,[11] which was represented by a linear combination of PxPy (85%) and s-like (15%) orbitals with a tip relaxation given by the AFM simulation mentioned above.

Dyson Orbitals

To interpret dI/dV maps obtained for molecules with a strong multi-reference character, it is essential to go beyond the single-determinant molecular orbital framework provided by DFT calculations. Therefore, we utilized the many-body concept of Dyson orbitals to simulate the dI/dV maps for one electron removal and adding process in STM.[12] Dyson orbitals describe the processes of electron removal and addition during tunneling. In the limit where the one-electron approximation is valid, Dyson orbitals coincide with the canonical molecular orbitals. However, in molecules with a strong radical character the Dyson orbitals consist of linear combinations of the canonical orbitals, making the many-body picture an essential tool for the comparison of theory and experiment.

In our case, we construct the Dyson orbitals from the CASCI calculations for the neutral system and the charged system. Let us define $|\Psi_0\rangle$ as the Ground State of the neutral system, and let us call N to its number of electrons. Then we calculate the wavefunctions for the positively and negatively charged

systems: $|\Psi_{+,j}\rangle, |\Psi_{-,j}\rangle$, with j labels the states (ground states and excited states) of the charged systems. Then we can compute the overlaps

$$\varphi_{+,\mathrm{j}}(x) = \sqrt{N+1}\int \Psi_0(x_1,\cdots,x_{12})\Psi_{+,j}(x,x_1,\cdots,x_{12})dx_1\cdots dx_{12},$$

and

$$\varphi_{-,\mathrm{j}}(x) = \sqrt{N}\int \Psi_0(x,x_1,\cdots,x_{11})\Psi_{-,j}(x_1,\cdots,x_{11})dx_1\cdots dx_{11}.$$

In this work, we construct these orbitals for $j = 0,1$ to inspect the PIR, PIR-1, NIR, NIR+1 resonances shown in Figure S7.

Natural Orbitals

In order to unveil the radical character of the different coupled-triangulenes dimers we use the concept of Natural Orbitals. These orbitals are the ones that maximize the electronic occupation of the system[13] and are obtained by diagonalizing the one-particle reduced density matrix. For the multirreferential Ground State $|\Psi_0\rangle$ the matrix elements are given by

$$\rho_{jk} = \sum_{\sigma} \langle\Psi_0|\hat{C}^{\dagger}_{j\sigma}\hat{C}_{k\sigma}|\Psi_0\rangle,$$

where the indices j,k run over the active orbitals and the $\hat{C}^{\dagger}_{j\sigma}(\hat{C}_{j\sigma})$ are the creation (anhilation) operators in orbital j with spin σ. The eigenfunctions of this matrix correspond to the Natural Orbitals and their eigenvalues represent the occupation of each orbital. In this way, each single occupation of an orbital will correspond to a radical in the system. In Fig. S9 below we present the results of these analysis for **Dimers 1, 2, 2'A** and **2'B** at the Hubbard-CASCI level of theory.

Natural Transition Orbitals (NTO)

We have calculated the NTOs[14] for the spin-flip process to capture the spatial variation of the d*I*/d*V* maps corresponding to IETS spin excitation maps (see Figure S7). We build the matrix of the spin-flip operator corresponding to transition from the singlet to the triplet ground state. NTO orbitals are obtained from the diagonalization of the matrix $TT^{\dagger}$, where the matrix T is given by elements

$$T_{jk} = \langle\Psi_{\mathrm{triplet}}|\hat{C}^{\dagger}_{j\uparrow}\hat{C}_{k\downarrow}|\Psi_{\mathrm{singlet}}\rangle,$$

where the indices j,k run over the active orbitals and the $\hat{C}^{\dagger}_{j\uparrow}$ are the second-quantization operators associated to the employed active space. The eigenfunctions of such matrices provide the efficient channel for the spin excitation, whose corresponding d*I*/d*V* map is in good agreement with the acquired experimental signal for dimer 1 (see Figure 2h in the main text and Fig, S6b below for the simulated map).

Hubbard model

We have employed the Hubbard model to study the electronic structure of **Dimers 1, 2, 2'A** and **2'B** . The Hubbard model is known to perform excellently for benzenoid hydrocarbons , and we have explored at the CASCI level of theory the radicality and spectra of the structures as functions of the angles. We thus start from the hamiltonian

$$\hat{H} = -\sum_{i,j,\sigma} t_{i,j}\left(\hat{c}^{\dagger}_{i\sigma}\hat{c}_{j\sigma} + h.c.\right) + U\sum_{i,j,\sigma}\hat{n}_{i\uparrow}\hat{n}_{i\downarrow}, \quad (1)$$

with i labeling the pz orbitals of the carbon atoms, $\hat{n}_{i\sigma} = \hat{c}^{\dagger}_{i\sigma}\hat{c}_{i\sigma}$, $U = 3.8eV$ and the $t_{i,j} = -2.8eV$, except for the atoms i, j in dimers **1** and **2** which are at an angle, α, in which case we set $t_{i,j} = -2.8\cos(\alpha)$. The reason for this is that we are modeling the $\pi -$ conjugated system given by the pz orbitals. Each pz orbital, centered at its nuclei, is described by a function, $\phi(\mathbf{r}) \approx e^{-\lambda r}\frac{z}{r}$, so that the overlap of two pz orbitals at an angle is modified by the factor $\cos(\alpha)$.[15] We have studied the spin excitation as well as the radical character of these molecules with CASCI for this model Hamiltonian. Both for dimers **1** and **2'A, 2'B**, where weh ave experimental information about the spin excitation, we observe an excellent agreement with the theory. In Fig. S9 we present the radical characters of these molecules in their relaxed geometries, and in Fig. 1 of the main text we present the geometry-dependent spin excitation.

SPIN MODELS

We have fitted the electronic structure of the two **Dimers 1** and **2** to a Heisenberg $S = 1/2$ spin hamiltonians of the general form:

$$\hat{H}_M = -J_T\left(\vec{\hat{S}}_1\cdot\vec{\hat{S}}_2 + \vec{\hat{S}}_3\cdot\vec{\hat{S}}_4\right) + J'(\alpha,\beta)\left(\vec{\hat{S}}_1\cdot\vec{\hat{S}}_3 + \vec{\hat{S}}_2\cdot\vec{\hat{S}}_4\right) + J''(\alpha,\beta)\left(\vec{\hat{S}}_1\cdot\vec{\hat{S}}_4 + \vec{\hat{S}}_2\cdot\vec{\hat{S}}_3\right). \quad (2)$$

Here $J_T = 400meV$ (ferromagnetic exchange) reproduces the triplet-singlet excitation energy in the $S = 1$ triangulene, which is obtained from a Hubbard-CASCI(4,4) calculation. The antiferromagnetic $J'(\alpha,\beta), J''(\alpha,\beta)$ are parametrized as:

Dimer 1

$$J'(\alpha,\beta) = 35\cos(\alpha)$$
$$J'(\alpha,\beta) = 35\cos(\beta)$$

and

Dimer 2

$$J'(\alpha,\beta) = J''(\alpha,\beta) = 18\cos(\alpha)\cos(\beta)\cos(\alpha-\beta).$$

This simple spin model reproduces very well the results of the Hubbard model. As shown in Figure S9, the spin excitation is well described as a function of the angles with the Heisenberg hamiltonian in eq. (2). This allows us to understand **Dimers 1** and **2** as four delocalized molecular spins with anti-ferromagnetic inter-molecular exchange parametrized with the rotation angles of the para-biphenyl units.

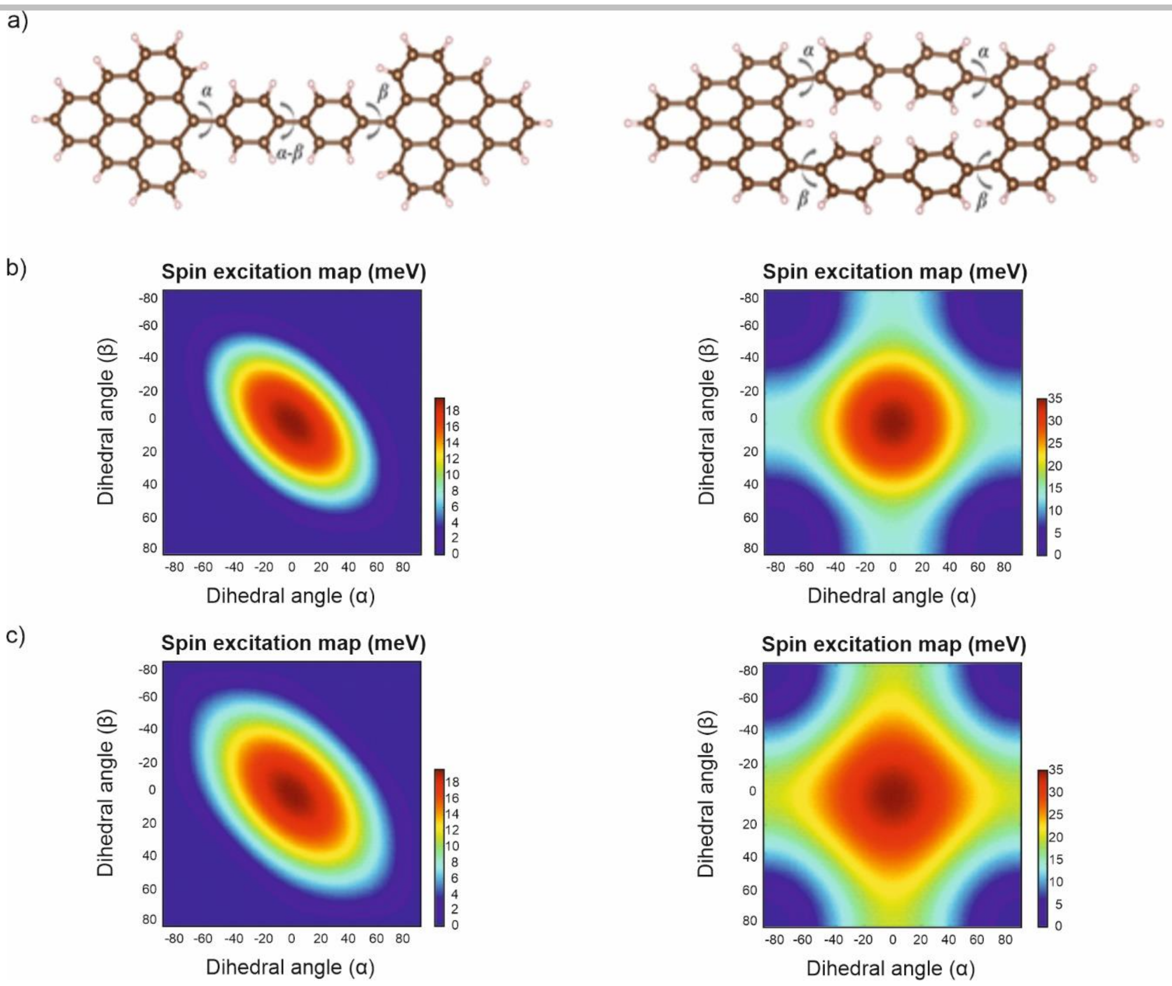

Figure S1. a) Reference planar structures for **Dimers 1** and **2**, b) spin excitation maps, as shown in Figure 1 in the main text, from Hubbard-CASCI(4,4) and c) spin excitation maps obtained from the spin hamiltonian [eq. (2)] with its correspondent parametrizations.

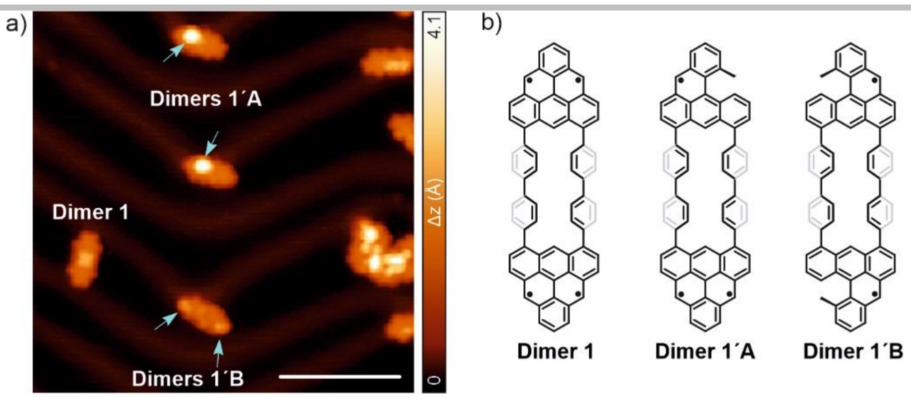

Figure S2. Sublimation of **1** onto an Au(111) surface held at 200 °C. a) Overview STM image showing the formation of Dimer 1 coexisting with dimers presenting some bright protrusions attributed to unreacted methyls and ill-defined nanostructures. b) Chemical sketches of **Dimer 1** and the tentative ones attributed to **Dimers 1´A** and **B**. Scanning parameters: V_b = 0.5 V, I_t = 50 pA, scale bar = 5 nm.

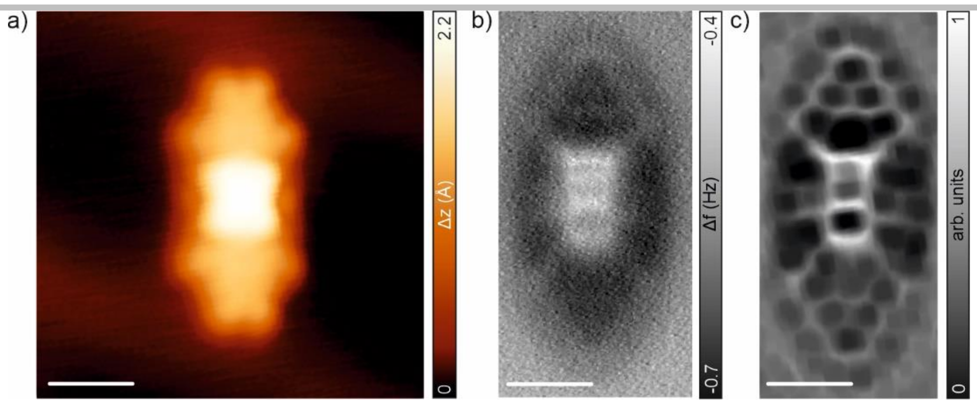

Figure S3. **Dimer 1** onto Au(111). a) STM image showing **Dimer 1** located at the fcc region of the Au(111) substrate. b) Constant-height frequency-shift image of **Dimer 1** acquired at a closer tip distance as the one depicted in Figure 2d. c) Laplace filtered image of (b) where the triangulene termini can be discerned. Scanning parameters: V_b = 0.5 V, I_t = 50 pA, scale bar = 0.5 nm.

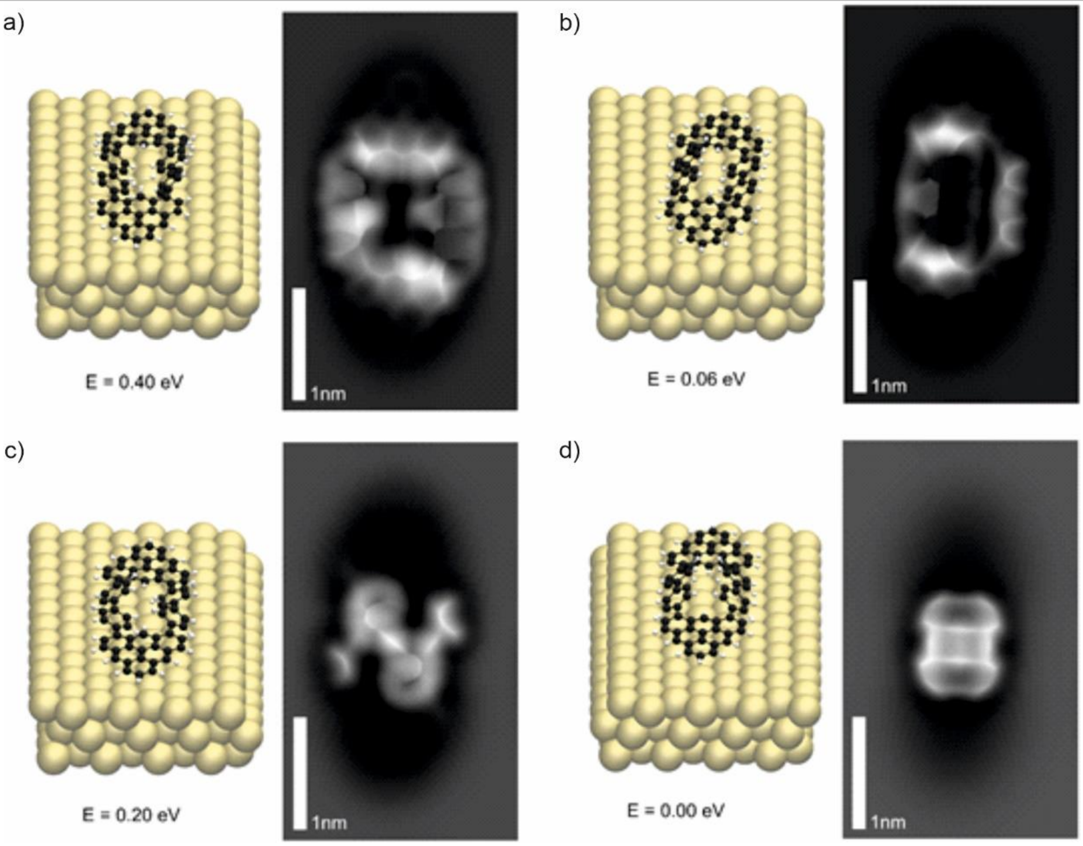

Figure S4. Four different ring alternation of the inner benzene rings of **Dimer 1**, relaxed with DFT, relative energies of the four absorption configurations and simulated AFM images. a-d) DFT geometries and simulated nc-AFM images showing the three discarded alternatives and the most energetically favorable structure panel (d), which was found in good agreement with the experimental results.

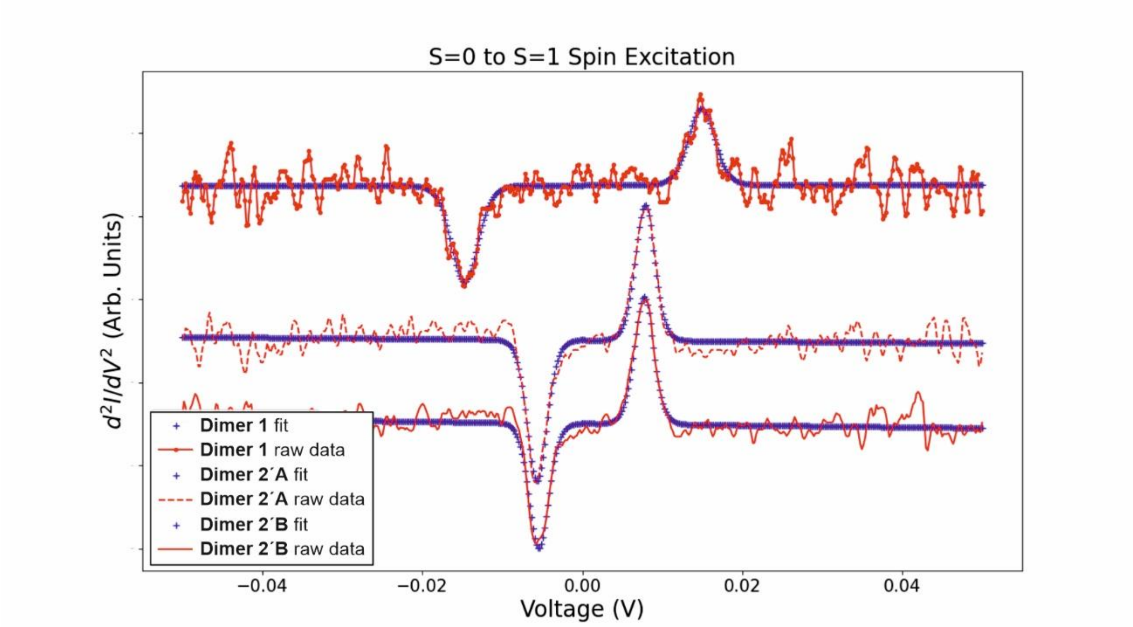

Figure S5. Fittings of the spectral features shown for Dimer 1, Dimer 2´A and Dimer 2´B in Figures 2g and 3f. The exact value obtained from the fittings (see methods for more details) is **Dimer 1**: 14.8 mV, **Dimer 2´A**: 6.5 mV, **Dimer 2´B**: 6.7 mV. The statistical uncertainties of the fits are of the order of 0.02 mV. To determine our experimental error, we have performed fits on several curves leading to these values for the spin-exitation energies: **Dimer 1**: 14.7 ± 0.2 mV, **Dimer 2´A**: 6.4 ± 0.2 mV and **Dimer 2´B**: 6.7 ± 0.1 mV. The effective temperature used to obtain the experimental peak was 19 ± 3 K. This includes all sources of broadening such as the lock-in modulation, intrinsic line-width and base temperature of the system. Spectra are offset for better visibility.

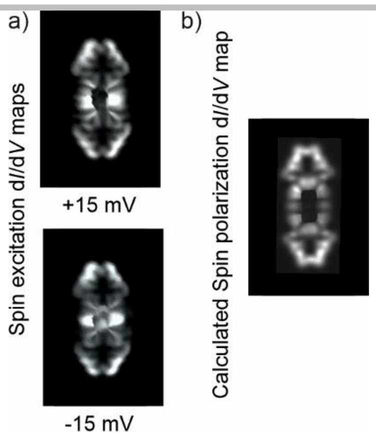

Figure S6. Comparison between experimental and theoretical spin polarization for **Dimer 1**. a) Constant-current d*I*/d*V* maps of **Dimer 1** at its spin excitation energies (I_t = 150 pA and V_{rms} = 10 mV). b) D*I*/d*V* map for a relaxed CO tip constructed with the PP-SPM code from the Natural Transition Orbitals for the singlet-triplet transition (see section 2 for details).

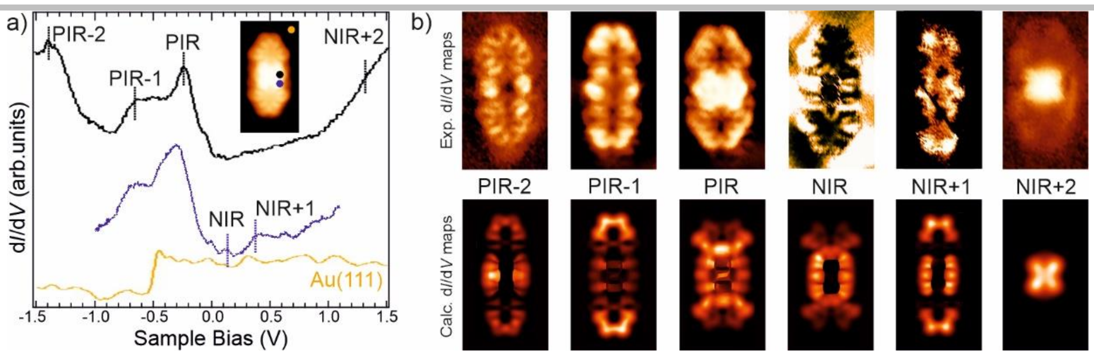

Figure S7. Frontier molecular orbitals of Dimer 1. a) d*I*/d*V* spectrum acquired on **Dimer 1** at the positions indicated by the black and blue rounded mark in the inset STM image, revealing a PIR-NIR gap of ~0.4 eV, and reference spectrum taken on the bare Au(111) surface (orange rounded mark in the inset STM image). Constant-current differential conductance d*I*/d*V* maps (top panel) and corresponding Dyson-calculated LDOS maps (bottom panel) from CAS-CI(8,8) at the energetic positions corresponding to the frontier orbitals of **Dimer 1**. Experimental NIR and NIR+1 are tentatively found at V_b = 0.1 and 0.3 V, respectively, showing very weak peak intensity. Tunneling parameters for the d*I*/d*V* maps: PIR-2 (V_b = −1.40 V, I_t = 500 pA); PIR-1 (V_b = −0.65 V, I_t = 500 pA); PIR (V_b = −0.22 V, I_t = 500 pA), NIR (V_b = 0.15 V, I_t = 500 pA), NIR+1 (V_b = 0.30 V, I_t = 500 pA) and NIR+2 (V_b = 1.30 V, I_t = 500 pA).

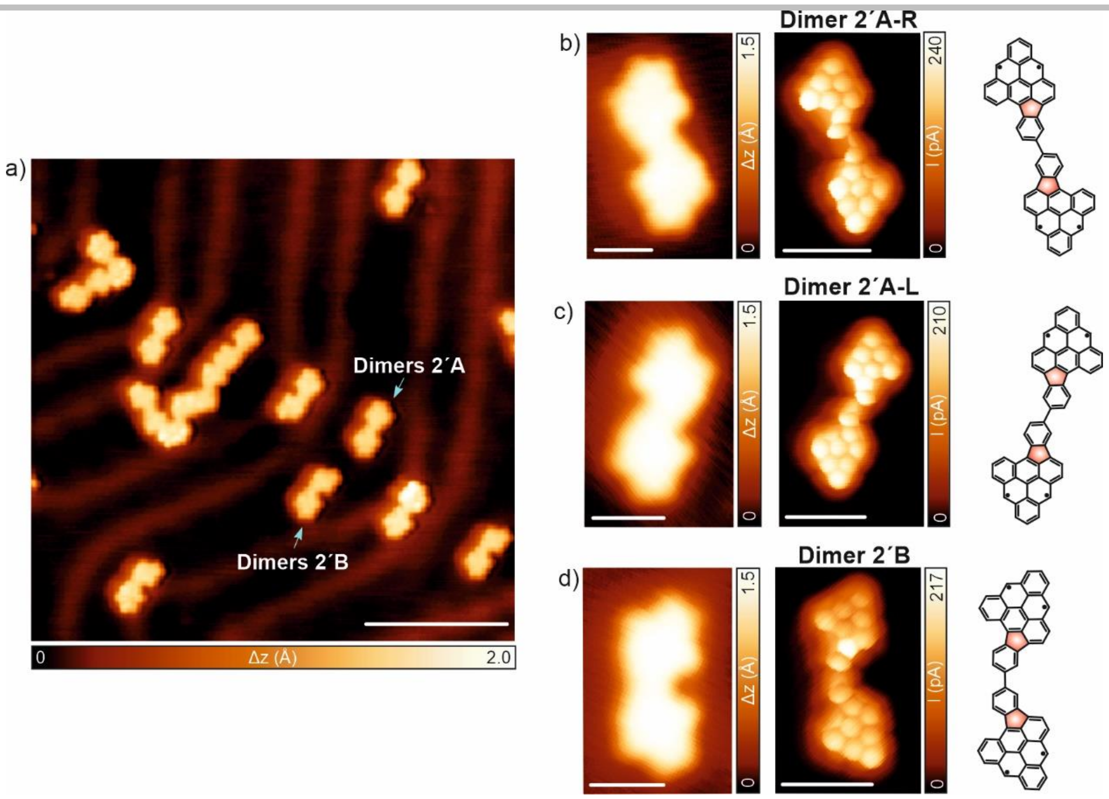

Figure S8. Prochiral (Dimer 2´A) and achiral (Dimer 2´B) observed on the Au(111) surface. a) Overview STM image after deposition of precursor **2** with the sample at 200 ºC. b,c) Constant-current, constant-height and chemical sketch of **Dimers 2´A**. The two enantiomers, named **Dimer 2´A-R** and **Dimer 2´A-L** are equally formed on the gold surface. d) Constant-current, constant-height and chemical sketch of **Dimer 2´B**. Such dimer presents a symmetry plane between the central benzene rings. Scanning parameters: V_b = 0.5 V, I_t = 50 pA, scale bars = 5 nm (a) and 1 nm (b-d). All the STM images were acquired using a CO-functionalized tip.

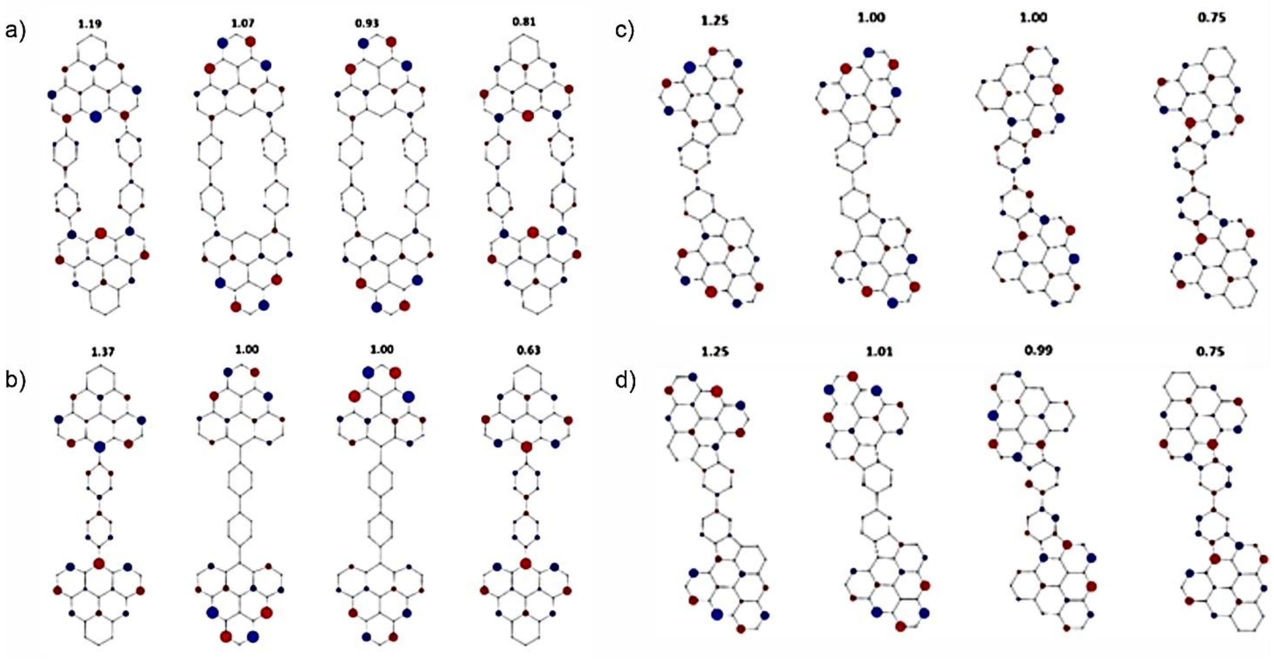

Figure S9. Natural orbitals with their electronic occupation from CAS-CI(4,4) for a) **Dimer 1** for the geometry shown in Fig. S4d) (relaxed geometry on a gold substrate); b) **Dimer 2,** corresponding also to the relaxed geometry on the substrate; c) **Dimer 2'A**; d) **Dimer 2'B**.

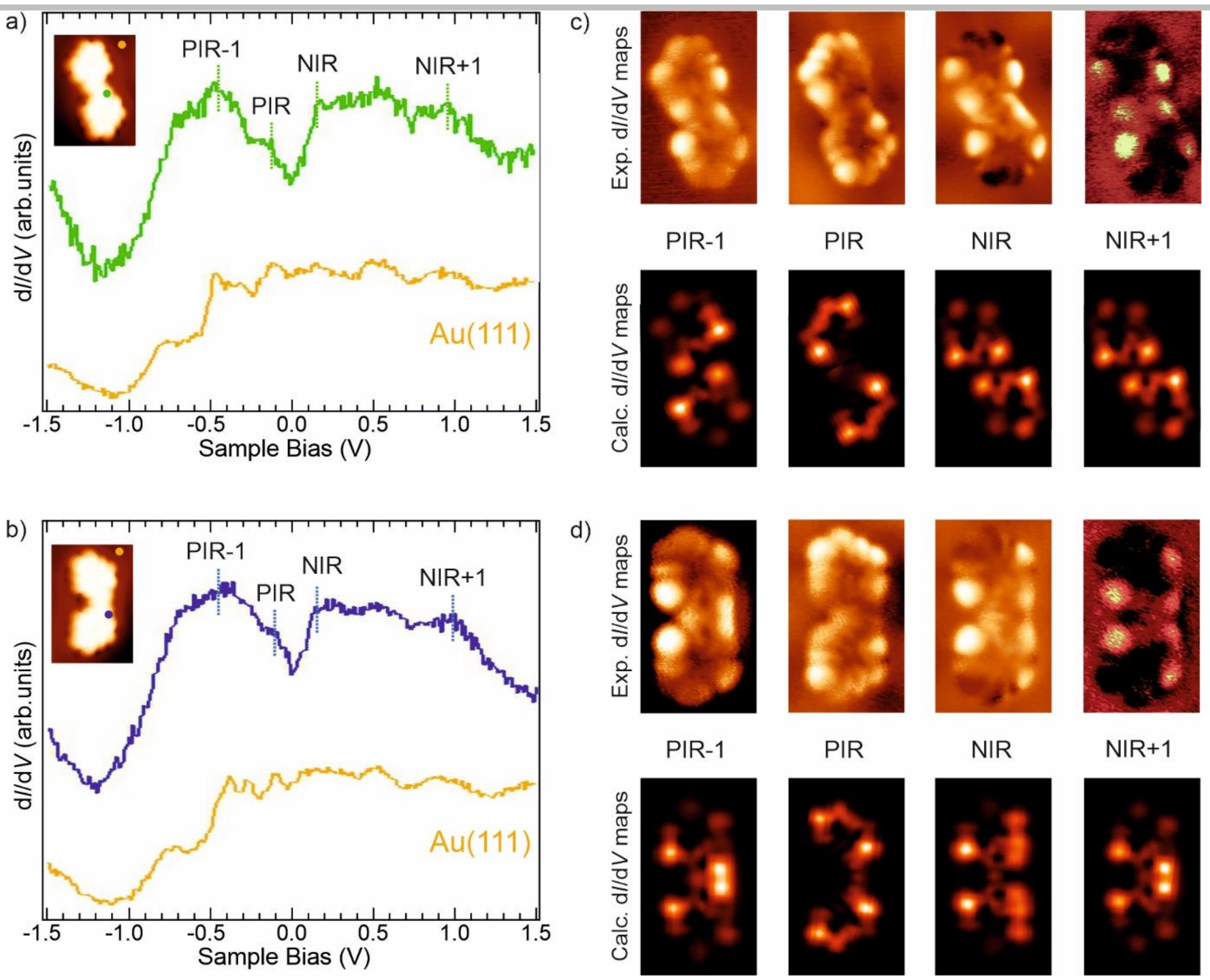

Figure S10. Frontier molecular orbitals assigned to the PIR-1, PIR, NIR and NIR+1 of Dimers 2´. a,b) d*I*/d*V* spectra acquired on both **Dimer 2´A** and **Dimer 2´B** at the positions indicated by the green and blue rounded marks in the inset STM images, revealing a PIR-NIR gap of ~0.3 eV, and reference spectrum taken on the bare Au(111) surface (orange rounded marks in the inset STM images). c,d) Constant-current differential conductance d*I*/d*V* maps (top panel) and corresponding Dyson-calculated LDOS maps (bottom panel) from CAS-CI(8,8) at the energetic positions corresponding to the frontier orbitals of **Dimer 2´A** and **Dimer 2´B**, respectively. Tunneling parameters for the d*I*/d*V* maps: PIR-1 (V_b = −0.45 V, I_t = 500 pA); PIR (V_b = −0.15 V, I_t= 500 pA); NIR (V_b = 0.15 V, I_t = 500 pA) and NIR+1 (V_b = 0.9 V, I_t = 500 pA).

3. General methods and materials

All the reagents were obtained from Sigma Aldrich, TCI, abcr, Alfa Aesar, Strem, fluorochem, and chempur. All these chemicals were used as received without further purification. All reactions dealing with air- or moisture-sensitive compounds were carried out in a dry reaction vessel under argon (Ar) atmosphere by using standard vacuum-line and Schlenk techniques. Anhydrous dichloromethane and toluene were obtained from MBRAUN MB-SPS-5 solvent purification system.

Thin layer chromatography (TLC) was performed on silica-coated aluminium sheets with a fluorescence indicator (TLC silica gel 60 F254, purchased from Merck KGaA).

Column chromatography was performed on silica (SiO_2, particle size 0.063 - 0.200 mm, purchased from VWR).

Nuclear magnetic resonance (NMR) spectra were recorded on a Bruker Avance III HD 300 spectrometer (300 MHz and 75.5 MHz for ^{1}H and ^{13}C respectively) or Bruker 400 spectrometer (400 MHz and 101 MHz for ^{1}H and ^{13}C respectively) using a 5 mm ^{1}H/^{13}C gradient probe at room temperature. $CDCl_3$ (δ (^{1}H) = 7.26 ppm, δ (^{13}C) = 77.16 ppm) or CD_2Cl_2 (δ (^{1}H) = 5.30 ppm, δ (^{13}C) = 54.0 ppm) was used as the solvent, lock and internal standard for ^{1}H and ^{13}C measurements.

The mass spectrometry analysis was performed on a Bruker Autoflex Speed MALDI-TOF MS (Bruker Daltonics, Bremen, Germany) using DCTB (*trans*-2-[3-(4-tert-Butylphenyl)-2-methyl-2-propenylidene]malononitrile) as matrix.

4. Synthetic procedures and characterization data

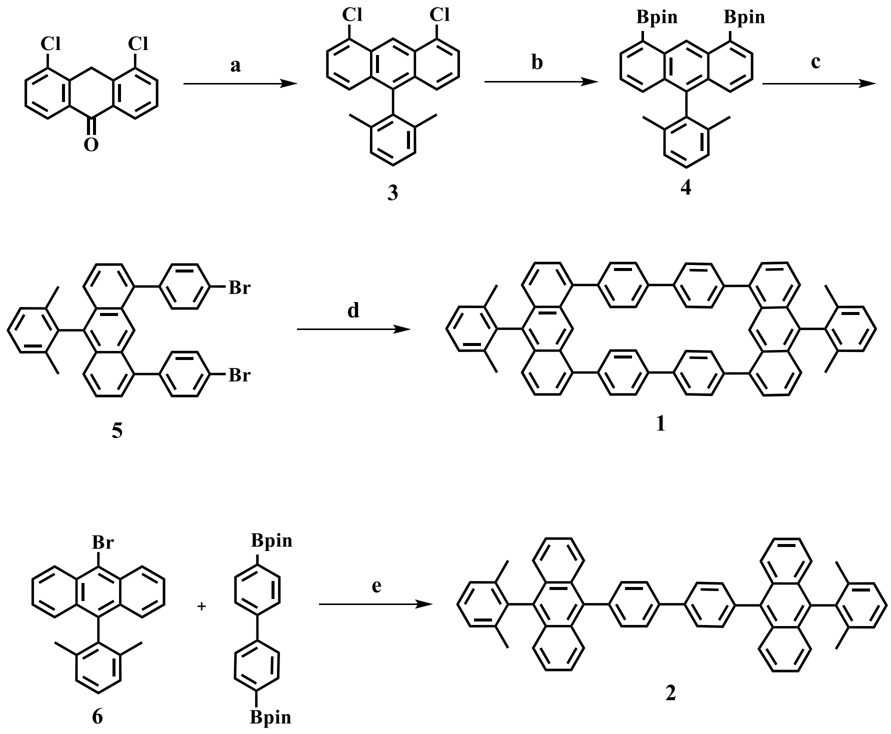

Figure S11. Synthetic route of 1 and 2. Reagents and conditions: a. (2,6-dimethylphenyl)magnesium bromide, Et_2O, rt, 12 h; b. Bis(pinacolato)diboron (B_2pin_2), SPhos Pd G3, KOAc, DMF, 130 °C , 48 h; c. $Pd(PPh_3)_4$, 1,4-dibromobenzene, K_2CO_3, toluene/H_2O, 80 °C, 24 h; d. $Ni(cod)_2$, 2,2'-bipyridyl,1,5-cyclooctadien (cod), toluene,

80 °C , 48 h; e. $Pd(PPh_3)_4$, K_2CO_3, toluene/H_2O, 80 °C , 24 h.

1,8-dichloro-10-(2,6-dimethylphenyl)anthracene (3)

To a solution of the commercially available 4,5-dichloroanthracen-9(10*H*)-one (5.0 g, 19.0 mmol) in dry Et_2O (40 mL) was added the (2,6-dimethylphenyl)magnesium bromide solution (1.0 M in THF, 57.0 mL, 3.0 eq, 57.0 mmol) at 0 °C under argon. The solution was stirred at room temperature overnight. The reaction mixture was quenched with aq. ammonium chloride, and the organic layer was extracted with ether. The organic solvents were removed under reduced pressure. Flash column chromatography on silica gel with hexane as the eluent afforded **3** as a white solid (3.1g, 8.8 mmol, 46%). ^{1}H NMR (CD_2Cl_2, 400 MHz, 298 K, ppm): δ 9.40 (s, 1H), 7.64 (d, J = 8 Hz, 2H), 7.42-7.34 (m, 3H), 7.29-7.25 (m, 4H), 1.71 (s, 6H); ^{13}C NMR (101 MHz, CD_2Cl_2) δ 138.1, 138.0, 137.4, 133.3, 131.3, 130.0, 128.8, 128.2, 126.7, 126.6, 125.8, 121.0, 20.3. HRMS (MALDI-TOF) m/z calcd. for $C_{22}H_{16}Cl_2$ $[M]^+$: 350.0629, found: 350.0687.

2,2'-(10-(2,6-dimethylphenyl)anthracene-1,8-diyl)bis(4,4,5,5-tetramethyl-1,3,2-dioxaborolane) (4)

A 25 mL Schlenk tube containing 1,8-dichloro-10-(2,6-dimethylphenyl)anthracene (**3**) (0.7 g, 2.0 mmol), SPhos Pd G3 (40.0 mg, 0.1 mmol), dried KOAc (0.6 g, 6.0 mmol), and bis(pinacolato)diboron (1.0 g, 4.0 mmol) was evacuated and filled with argon three times. Dry DMF (5.0 mL) was then transferred to the flask via syringe under argon. The mixture was stirred at 130 °C for 48 h. After cooling down to room temperature, water was added and the mixture was extracted with EtOAc. The combined organic layers were washed with brine, dried over anhydrous $MgSO_4$ and the organic solvents were removed under reduced pressure. The residue was purified by silica gel column chromatography (hexane/dichloromethane (10:1) to give **4** as a white solid (0.4 g, 0.7 mmol, 35%). ^{1}H NMR ($CDCl_3$, 300 MHz, 298 K, ppm): δ 9.82 (s, 1H), 8.13 (dd, J = 6.6, 1.5 Hz, 2H), 7.71 (d, J = 8.4 Hz, 2H), 7.37-7.27 (m, 3H), 7.25-7.20 (m, 2H), 1.68 (s, 6H), 1.50(s, 24H). ^{13}C NMR ($CDCl_3$, 75 MHz, 298 K, ppm): δ138.3, 137.8, 136.4, 136.2, 134.5, 129.2, 129.0, 127.5, 127.4, 127.2, 124.7, 83.7, 25.0, 20.0, C-B signal not observed. HRMS (MALDI-TOF) m/z calcd. for $C_{34}H_{40}B_2O_4$ $[M]^+$: 534.3113, found: 534.3116.

1,8-bis(4-bromophenyl)-10-(2,6-dimethylphenyl)anthracene (5)

A 50 mL Schlenk tube was charged with **4** (0.1 g, 0.2 mmol), 1,4-dibromobenzene (176.8 mg, 0.8 mmol), $Pd(PPh_3)_4$ (20.0 mg, 17.0 μmol) and K_2CO_3 (156.0 mg, 1.1 mmol). After purging with argon three times, degassed toluene (10.0 mL) and water (1.0 mL) were added to the solids. The mixture was then heated at 80 °C for 24 h under argon atmosphere. After cooling down to room temperature, water was added and the mixture was extracted with dichloromethane. The organic layer was dried over anhydrous $MgSO_4$ and the volatiles were evaporated. The solid residue was purified by silica gel column chromatography using hexane/dichloromethane (30:1) as eluent to give **5** as a white solid (65.1 mg, 0.1 mmol, 59%). ^{1}H NMR ($CDCl_3$, 300 MHz, 298 K, ppm): δ 8.45 (s, 1H), 7.59 (d, J = 8.4 Hz, 4H), 7.51-7.46

(m, 2H), 7.41-7.36 (m, 9H), 7.31-7.29 (m, 2H), 1.81 (s, 6H). ^{13}C NMR ($CDCl_3$, 75 MHz, 298 K, ppm): δ139.5, 139.4, 137.7, 136.2, 131.7, 131.3, 129.8, 129.6, 127.8, 127.6, 126.0, 125.7, 125.5, 123.2,121.54, 20.2, one carbon is overlaid. HRMS (MALDI-TOF) m/z calcd. for $C_{34}H_{24}Br_2$ $[M]^+$: 592.0224, found: 592.0235.

39,44-bis(2,6-dimethylphenyl)-10,13:14,17:27,30:31,34-tetraetheno-4,6:21,23-di(metheno)tetrabenzo[*a,d,n,q*][26]annulene (1)

A 50 mL Schlenk flask containing compound **5** (59.2 mg, 0.1 mmol) and 2,2'-bipyridyl (bpy) (44.0 mg, 0.3 mmol) was evacuated and refilled with argon for three cycles. Then, $Ni(cod)_2$ (72.0 mg, 0.3 mmol) was added, and the flask was evacuated and refilled with argon for another three cycles. Dry toluene (10.0 mL) and 1,5-cyclooctadien (cod) (0.1 mL) were transferred to the flask via syringe under argon at room temperature. The resultant mixture was allowed to stir for 0.5 h at room temperature and then heated at 80 °C for 48 h. After the reaction mixture was cooled to room temperature, the solvent was removed under vacuum and the residue was purified by silica gel column chromatography using hexane/dichloromethane (4:1) as eluent to give precursor **1** as a white solid (5.2 mg, 0.006 mmol, 12%). ^{1}H NMR ($CDCl_3$, 300 MHz, 298 K, ppm): δ 8.71 (s, 2H), 7.78 (d, J = 8.1 Hz, 8H), 7.61 (d, J = 8.1 Hz, 8H), 7.52-7.39 (m, 14H), 7.33 (d, J = 7.2 Hz, 4H), 1.87 (s, 12H). Due to the low concentration, ^{13}C NMR measurement could not be carried out. HRMS (MALDI-TOF) m/z calcd. for $C_{68}H_{48}$ $[M]^+$: 864.3756, found: 864.3749.

4,4'-bis(10-(2,6-dimethylphenyl)anthracen-9-yl)-1,1'-biphenyl (2)

A flask containing compound 9-bromo-10-(2,6-dimethylphenyl)anthracene (**6**), which was prepared following a previously reported procedure[16] (125.0 mg, 0.3 mmol), the commercially available 4,4'-bis(4,4,5,5-tetramethyl-1,3,2-dioxaborolan-2-yl)-1,1'-biphenyl (46.8 mg, 0.1 mmol), $Pd(PPh_3)_4$ (20.0 mg, 17.0 µmol) and K_2CO_3 (52.6 mg, 0.4 mmol) in toluene (10.0 mL) and H_2O (1.0 mL) was degassed for 30min. The mixture was then heated at 80 °C for 24 h under argon atmosphere. After cooling down to room temperature, water was added and the mixture was extracted with dichloromethane. The organic layer was dried over anhydrous $MgSO_4$ and the volatiles were evaporated. The residue was purified by silica gel column chromatography using hexane/dichloromethane (10:1) as eluent to give precursor **2** as a white solid (49.0 mg, 69.0 µmol, 60%). ^{1}H NMR ($CDCl_3$, 300 MHz, 298 K, ppm): δ 8.06 (d, J = 8.1 Hz, 4H), 7.88 (d, J = 7.8 Hz, 4H), 7.69 (d, J = 8.1 Hz, 4H), 7.54 (d, J = 7.5 Hz, 4H), 7.43-7.30 (m, 14H), 1.85 (s, 12H). ^{13}C NMR ($CDCl_3$, 75 MHz, 298 K, ppm): δ139.8, 138.3, 137.9, 137.8, 136.4, 135.8, 132.1, 130.2, 129.2, 127.7, 127.5, 127.3, 127.1, 126.0, 125.4, 125.2, 20.2. HRMS (MALDI-TOF) m/z calcd. for $C_{56}H_{42}$ $[M]^+$: 714.3287, found: 714.3198.

MALDI-TOF mass spectra

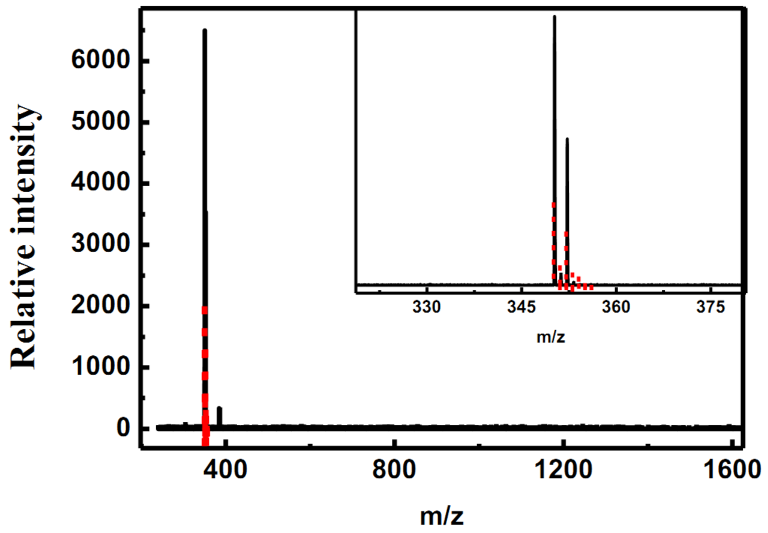

Figure S12. MALDI-TOF-MS spectrum (black) and simulated data (red) for **3**.

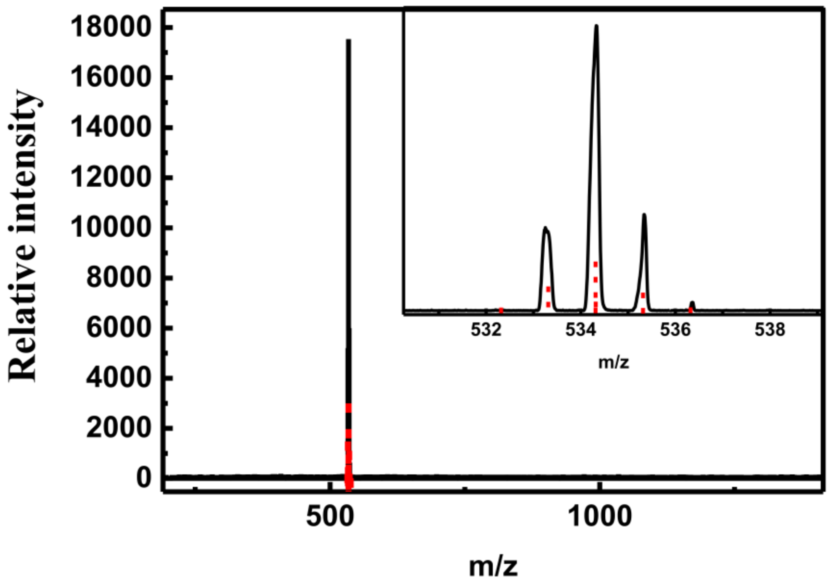

Figure S13. MALDI-TOF-MS spectrum (black) and simulated data (red) for **4**.

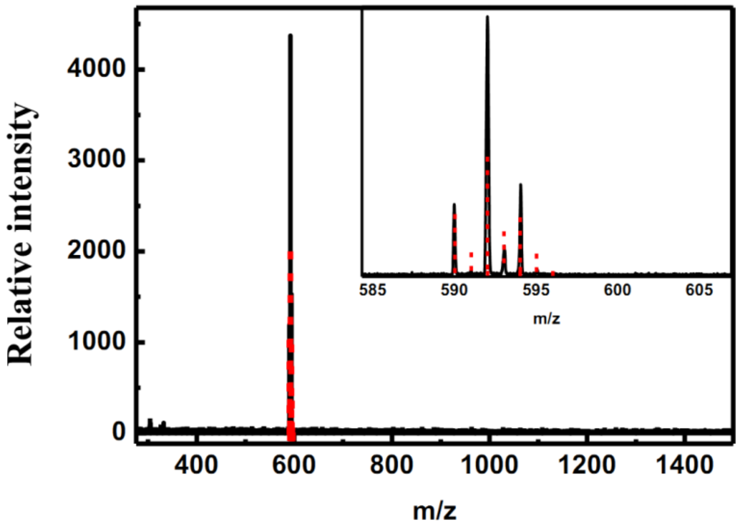

Figure S14. MALDI-TOF-MS spectrum (black) and simulated data (red) for **5**.

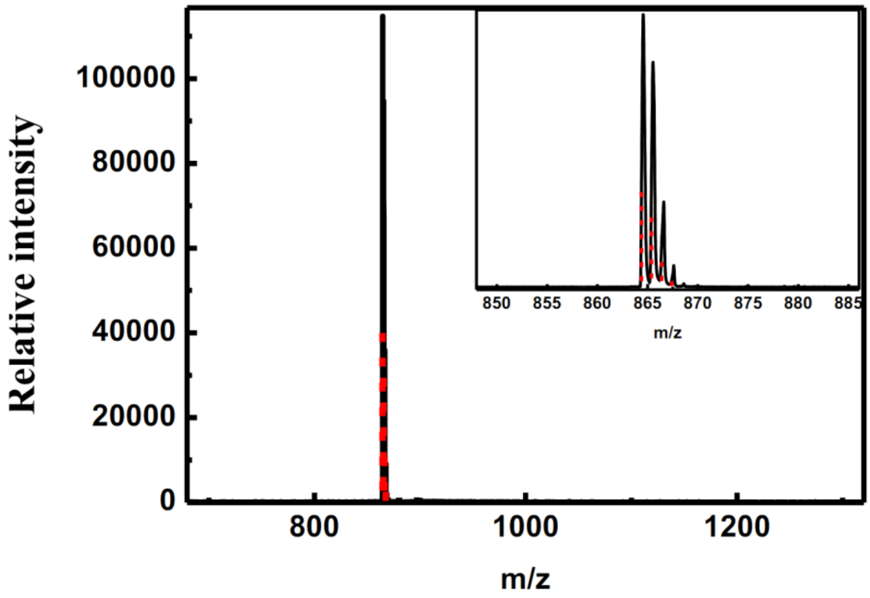

Figure S15. MALDI-TOF-MS spectrum (black) and simulated data (red) for **1**.

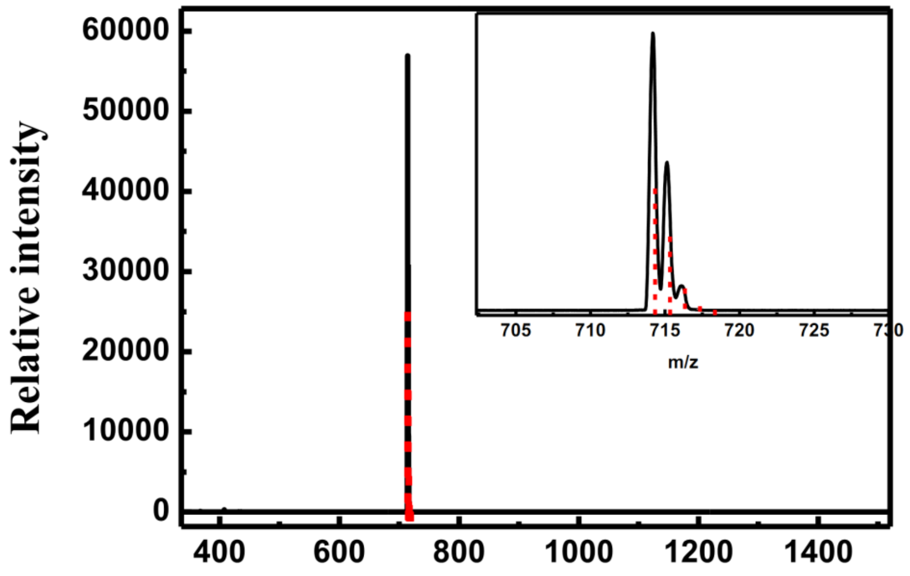

Figure S16. MALDI-TOF-MS spectrum (black) and simulated data (red) for **2**.

^{1}H NMR and ^{13}C NMR spectra for new compounds

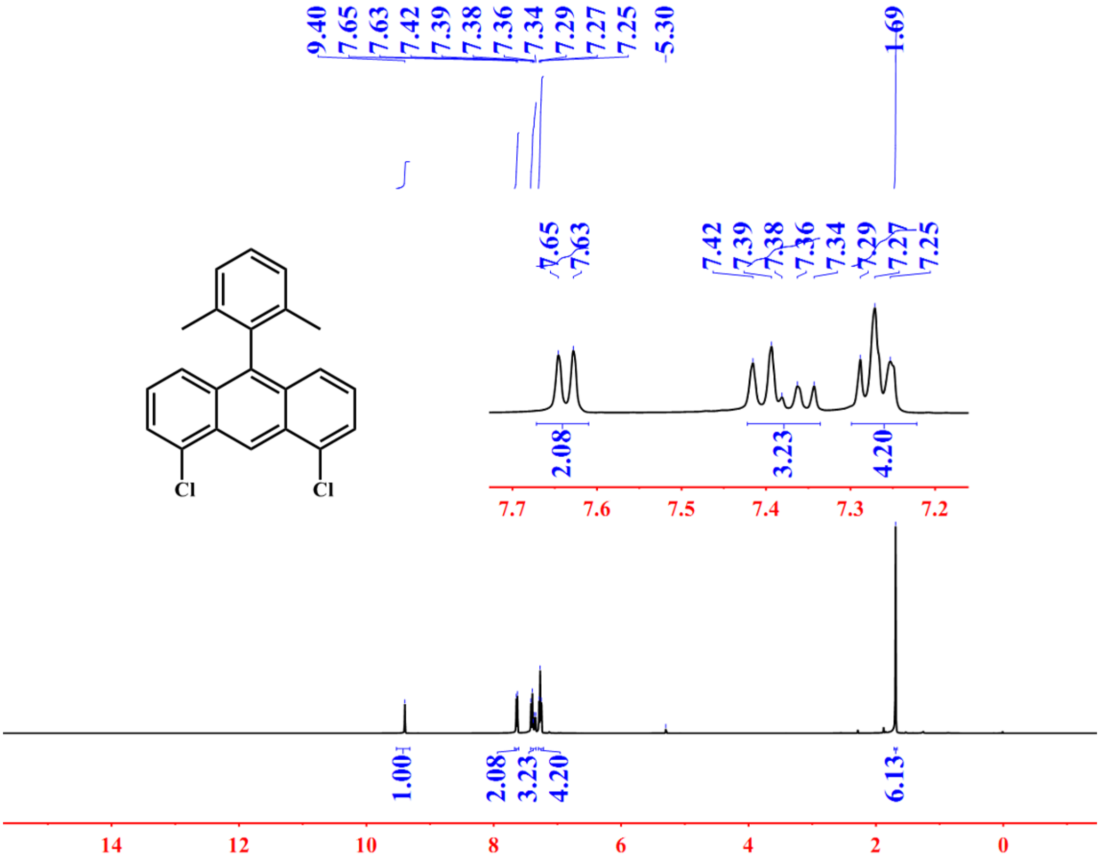

Figure S17. ^{1}H NMR spectrum of **3**.

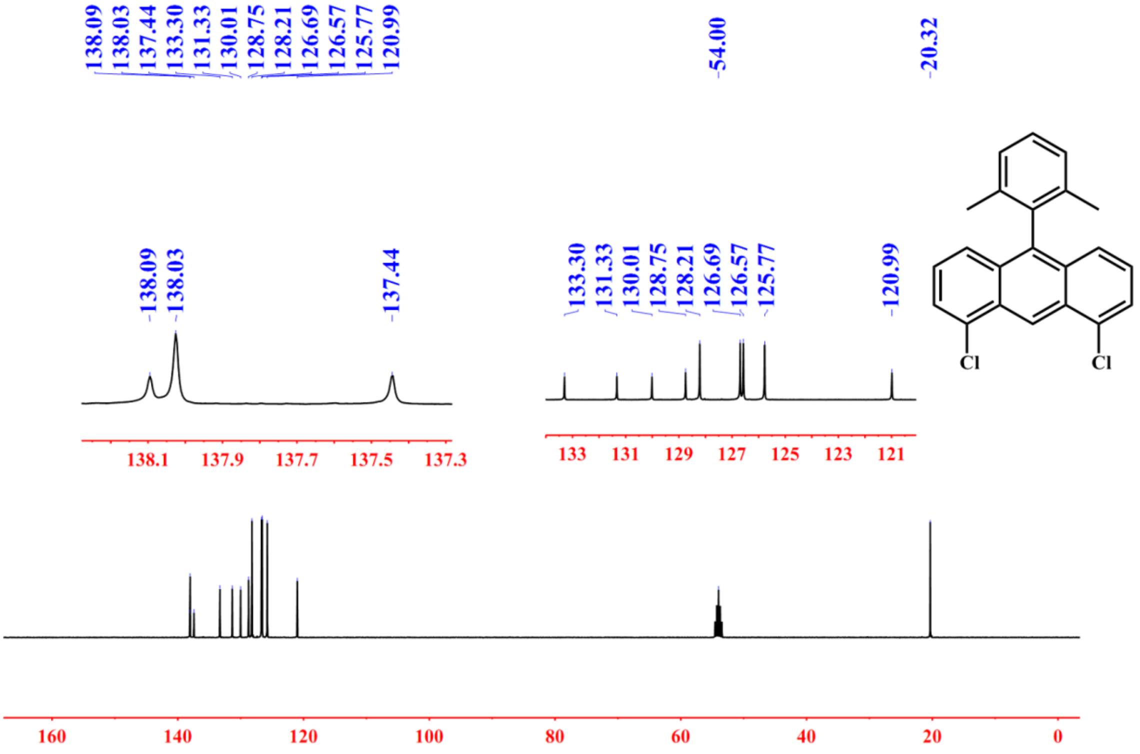

Figure S18. ^{13}C NMR spectrum of **3**.

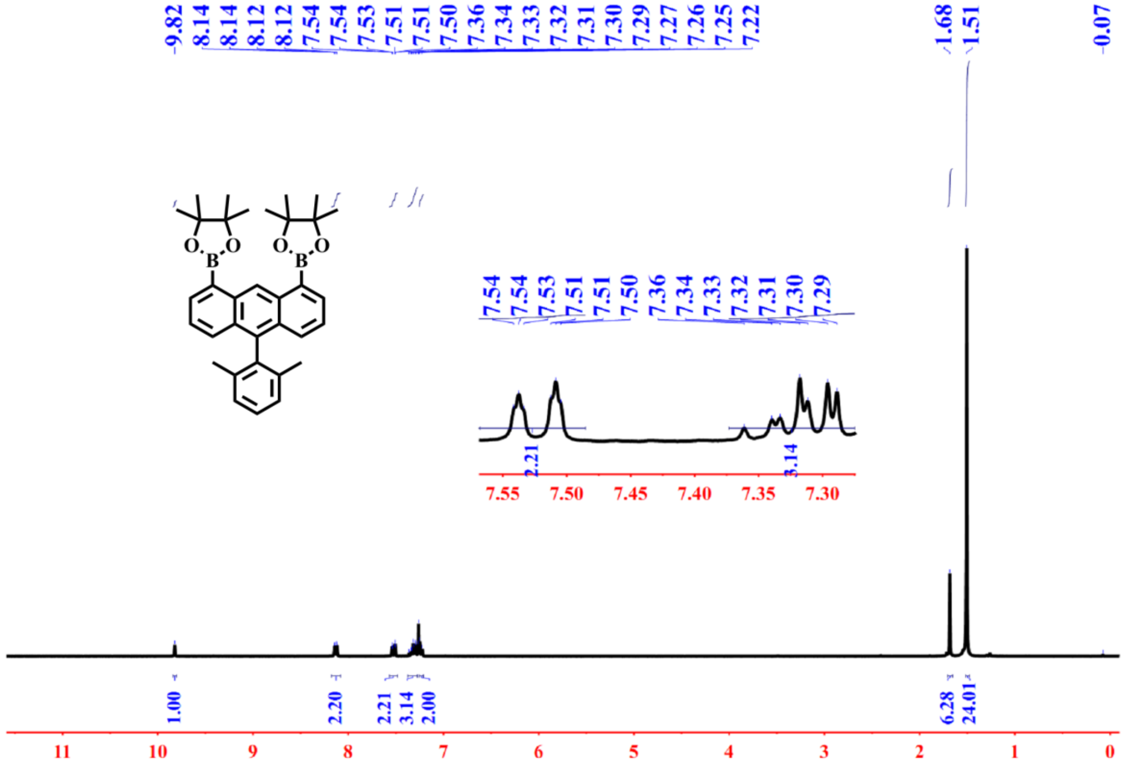

Figure S19. ^{1}H NMR spectrum of **4**.

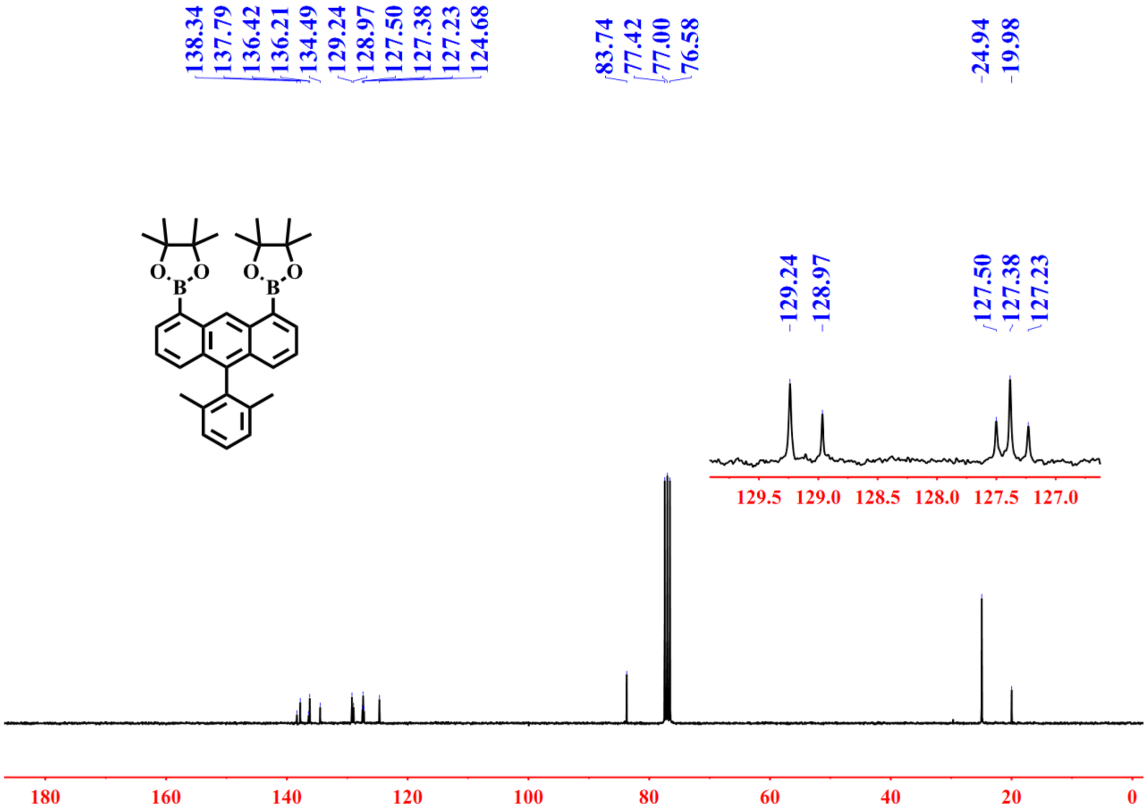

Figure S20. ^{13}C NMR spectrum of **4**.

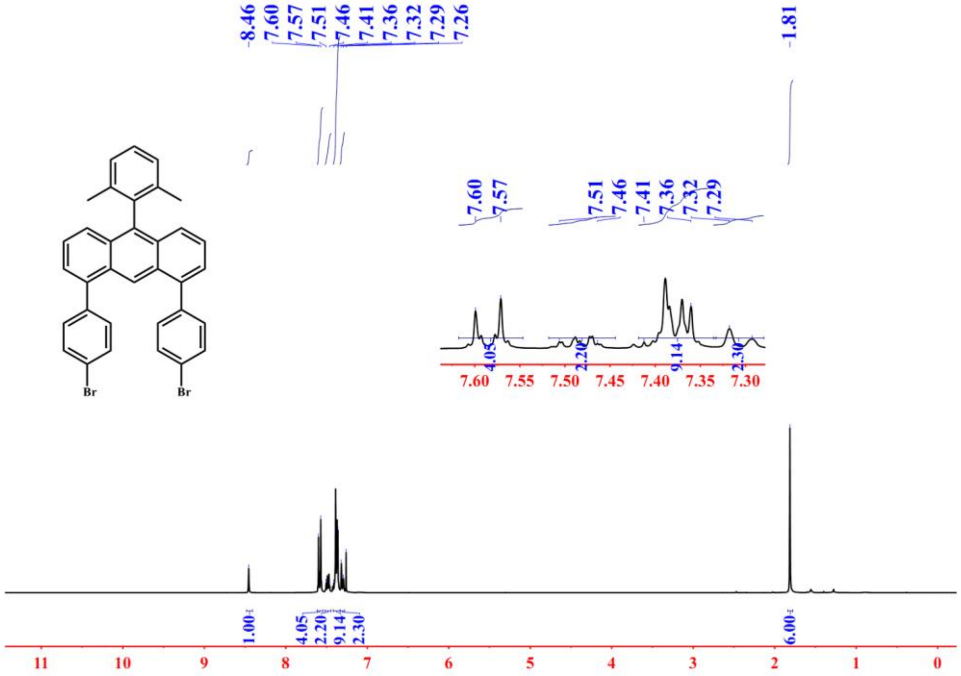

Figure S21. ^{1}H NMR spectrum of **5**.

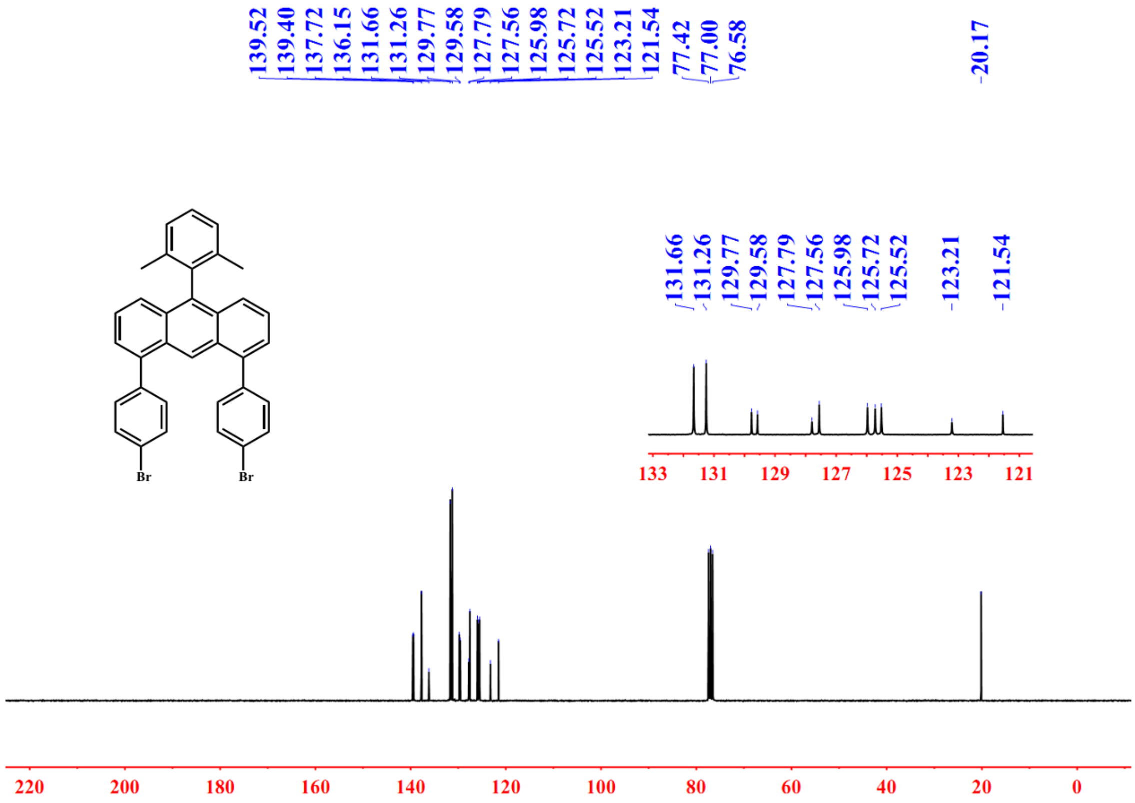

Figure S22. 13C NMR spectrum of **5**.

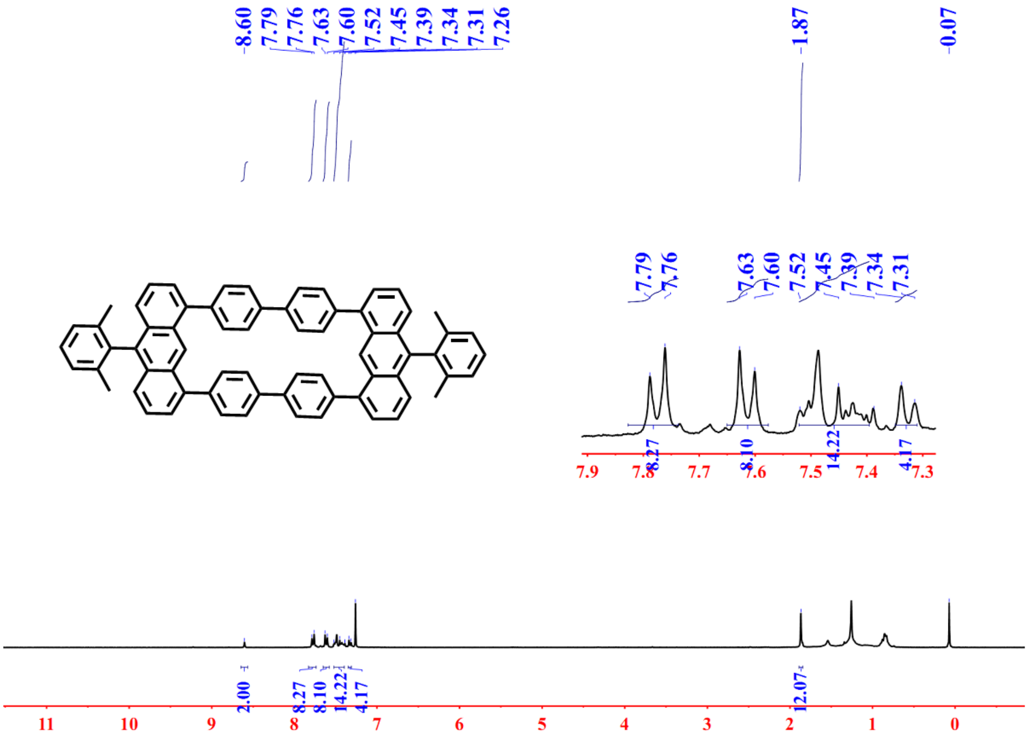

Figure S23. ^{1}H NMR spectrum of **1**.

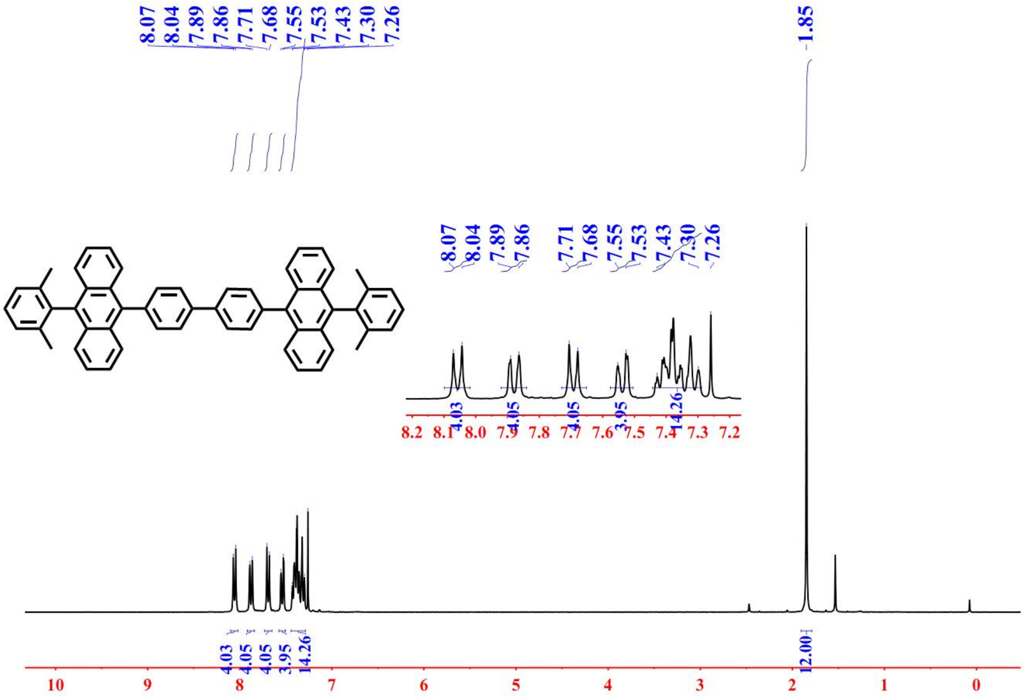

Figure S24. ^1H NMR spectrum of **2**.

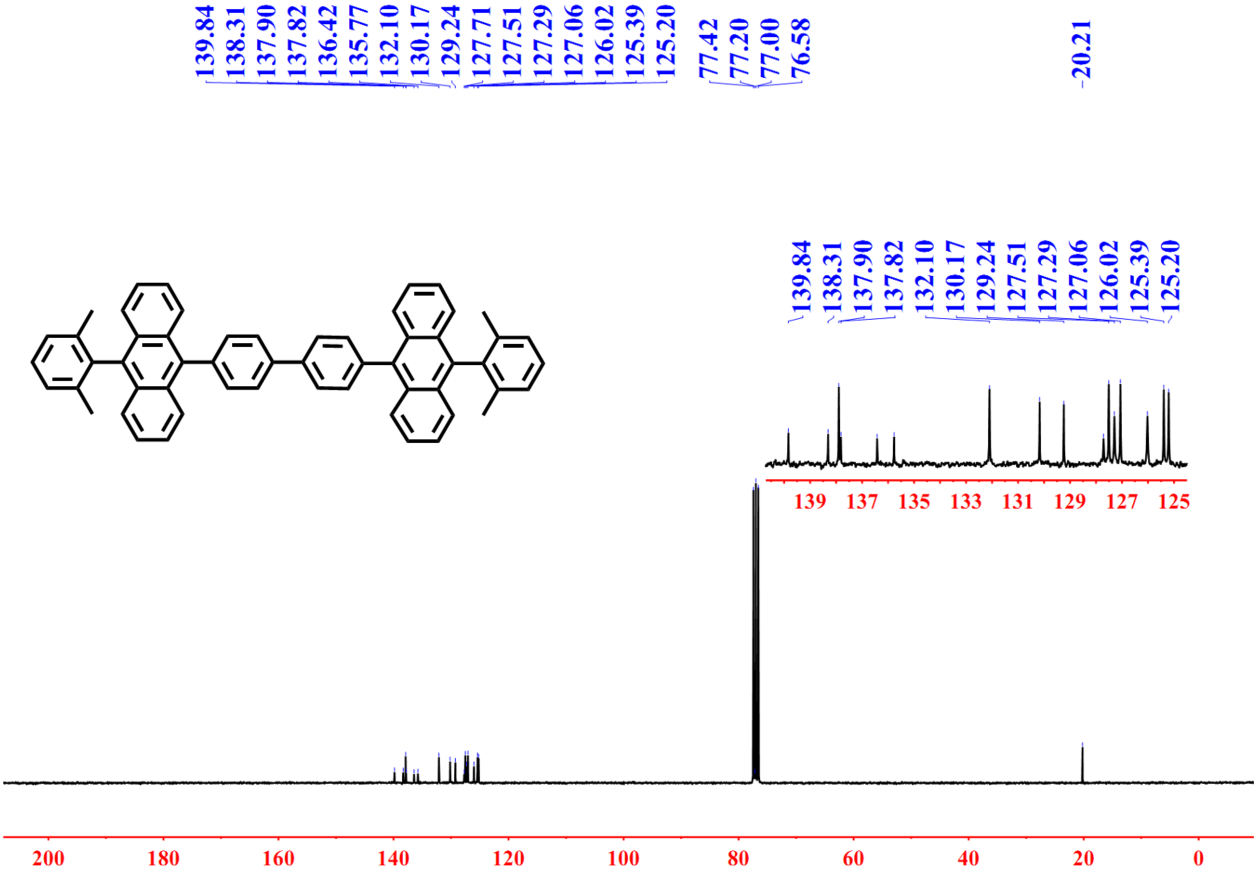

Figure S25. ^{13}C NMR spectrum of **2**.

5. References

[1] L. Bartels, G. Meyer, K.-H. Rieder, D. Velic, E. Knoesel, A. Hotzel, M. Wolf, G. Ertl, *Phys. Rev. Lett.* **1998**, *80*, 2004–2007.
[2] *Appl. Phys. Lett.* **2000**, *76*, 1470–1472.
[3] L. Gross, F. Mohn, N. Moll, P. Liljeroth, G. Meyer, *Science* **2009**, *325*, 1110–1114.
[4] M. Ternes, *New J. Phys.* **2015**, *17*, 063016.
[5] M. Gruber, A. Weismann, R. Berndt, *J. Phys. Condens. Matter* **2018**, *30*, 424001.
[6] I. Horcas, R. Fernández, J. M. Gómez-Rodríguez, J. Colchero, J. Gómez-Herrero, A. M. Baro, *Rev. Sci. Instrum.* **2007**, *78*, 013705.
[7] V. Blum, R. Gehrke, F. Hanke, P. Havu, V. Havu, X. Ren, K. Reuter, M. Scheffler, *Comput. Phys. Commun.* **2009**, *180*, 2175–2196.
[8] J. P. Perdew, K. Burke, M. Ernzerhof, *Phys. Rev. Lett.* **1996**, *77*, 3865–3868.
[9] A. Tkatchenko, M. Scheffler, *Phys. Rev. Lett.* **2009**, *102*, 073005.
[10] N. Oinonen, A. V. Yakutovich, A. Gallardo, M. Ondráček, P. Hapala, O. Krejčí, *Comput. Phys. Commun.* **2024**, *305*, 109341.
[11] O. Krejčí, P. Hapala, M. Ondráček, P. Jelínek, *Phys. Rev. B* **2017**, *95*, 045407.
[12] J. V. Ortiz, *J. Chem. Phys.* **2020**, *153*, 070902.
[13] P.-O. Löwdin, *Phys. Rev.* **1955**, *97*, 1474–1489.
[14] R. L. Martin, *J. Chem. Phys.* **2003**, *118*, 4775–4777.
[15] G. Seifert, D. Porezag, Th. Frauenheim, *Int. J. Quantum Chem.* **1996**, *58*, 185–192.
[16] X. Zhao, Y. Ju, Y. Su, C. Tang, Q. Zeng, L. Feng, C. Wang, K. Müllen, Y.Tan, J*. Am. Chem. Soc.* **2023**, *145*, 19333–19337.
